# Human promoter genomic composition demonstrates non-random groupings that reflect general cellular function

**DOI:** 10.1186/1471-2105-6-259

**Published:** 2005-10-18

**Authors:** Markey C McNutt, Ron Tongbai, Wenwu Cui, Irene Collins, Wendy J Freebern, Idalia Montano, Cynthia M Haggerty, GVR Chandramouli, Kevin Gardner

**Affiliations:** 1 The Advanced Technology Center, Laboratory of Receptor Biology and Gene Expression, National Cancer Institute, Bethesda, Maryland 20892-4605, USA; 2 The University of Texas Southwestern Medical Center at Dallas, TX, USA; 3 Bristol-Myers Squibb, Syracuse, NY, USA

## Abstract

**Background:**

The purpose of this study is to determine whether or not there exists nonrandom grouping of cis-regulatory elements within gene promoters that can be perceived independent of gene expression data and whether or not there is any correlation between this grouping and the biological function of the gene.

**Results:**

Using ProSpector, a web-based promoter search and annotation tool, we have applied an unbiased approach to analyze the transcription factor binding site frequencies of 1400 base pair genomic segments positioned at 1200 base pairs upstream and 200 base pairs downstream of the transcriptional start site of 7298 commonly studied human genes. Partitional clustering of the transcription factor binding site composition within these promoter segments reveals a small number of gene groups that are selectively enriched for gene ontology terms consistent with distinct aspects of cellular function. Significance ranking of the class-determining transcription factor binding sites within these clusters show substantial overlap between the gene ontology terms of the transcriptions factors associated with the binding sites and the gene ontology terms of the regulated genes within each group.

**Conclusion:**

Thus, gene sorting by promoter composition alone produces partitions in which the "regulated" and the "regulators" cosegregate into similar functional classes. These findings demonstrate that the transcription factor binding site composition is non-randomly distributed between gene promoters in a manner that reflects and partially defines general gene class function.

## Background

Amidst a continuous bombardment of diverse stimuli from the external environment, metazoan organisms have adopted multiple strategies to respond specifically and decisively to a myriad of extracellular events. The biological map that determines this is encoded within the gene regulatory regions of the genome. Deciphering the inherent language in these encrypted codes is a major challenge of the post-genomic era. The search, retrieval and examination of the upstream regulatory sequences of eukaryotic genes coupled with empirical determination of their transcriptional regulatory function has yielded a wealth of potentially useful information relevant to the sequence-specific codes used to dynamically coordinate the spatial, temporal, and kinetic assembly of gene regulatory complexes at specific genes [1]. Cells must orchestrate this coordinated gene expression in order to efficiently execute the multitude of cellular programs that direct specific functions.

Essential components of controlling networks that modulate cellular programming are the regulatory sequences or transcription factor binding sites (TFBS). TFBSs comprise the basic unit of information stored within the upstream genomic regions located near the transcription start site (TSS) of most genes [1,2]. These typically 8–15 bp nucleotide sequences interact specifically with the DNA-binding domains of several hundred different transcription factors. Since it is widely accepted that the TFBS arrangement and composition of these upstream regulatory regions are the fundamental determinants of gene expression, many software applications and computational approaches have been developed to sort and identify TFBSs in the regulatory regions of genes determined to have similar patterns of expression [3-5]. One popular approach is based on a software algorithm that compares the potential binding site base frequencies against an established database of empirically determined nucleotide frequencies derived from published biological studies [5]. The resulting position weight matrixes (PWM) are then used to search for and characterize potential binding sites dependent on their statistical similarities to known TFBSs. A major goal of this approach is to analyze co-occurring TFBS frequencies in the regulatory regions of similarly regulated genes as a means of defining transcriptional pathways or networks that orchestrate the co-expression. Most biologists measure steady state RNA levels as an indicator of gene expression. Thus, the linkages between TFBS occurrence and gene expression will undoubtedly be imperfect due to the fact that: 1) steady-state levels of expressed mRNA are a combined result of both active transcription and mRNA turnover; 2) indirect regulation of transcription factors by post-translational modification or other transcriptional components is a common control mechanism in metazoan biology; and 3) most PWM libraries are derived from empirical data sets and therefore have limited inclusiveness [1,6-8]. Nonetheless, focused and global analysis of gene promoter composition has the potential of yielding important insight into gene regulation.

Recent efforts to define a common vocabulary to describe the function of all genes through the use of established Gene Ontology terms has provided a standardized approach of analyzing genes, clustered by any objective criteria, with respect to their cellular function [9,10]. Combining the analysis of gene promoter composition with gene ontology annotation provides a novel and innovative means through which linkages between gene regulatory networks and programs of cellular function can be identified and defined.

In this study, we analyzed the transcription factor binding site composition of 1400 bp promoter regions defined as 1200 bp upstream and 200 bp downstream of the transcription start site of 7,298 genes previously characterized in a recent microarray study of the kinetic patterns of gene expression in a mitogen-stimulated human leukemic T-cell line [11]. Though the composition of TFBSs in these 7,298 genes show very poor correlation with the measured global kinetic patterns of steady-state gene expression, independent partitional clustering of the TFBS composition within these 1400 bp regions "in silico" produced definable non-random gene groups for which distinct classes of ontology terms were found more frequently than expected by random chance. Moreover, analysis of the TFBSs that were most significant for distinguishing these gene groups revealed strong correlations between the ontology terms of the transcription factors predicted to bind the controlling gene regulatory regions and the ontology terms of the clustered genes themselves; thus, establishing a functional link defined by the ontology of the regulated gene and that of its regulators (TFBS-associated transcription factors). Refinement of this approach may provide a general means of defining the regulatory genomic templates upon which transcriptional networks are integrated to control specific programs of gene expression and cellular behavior.

## Results

**Figure 1:**
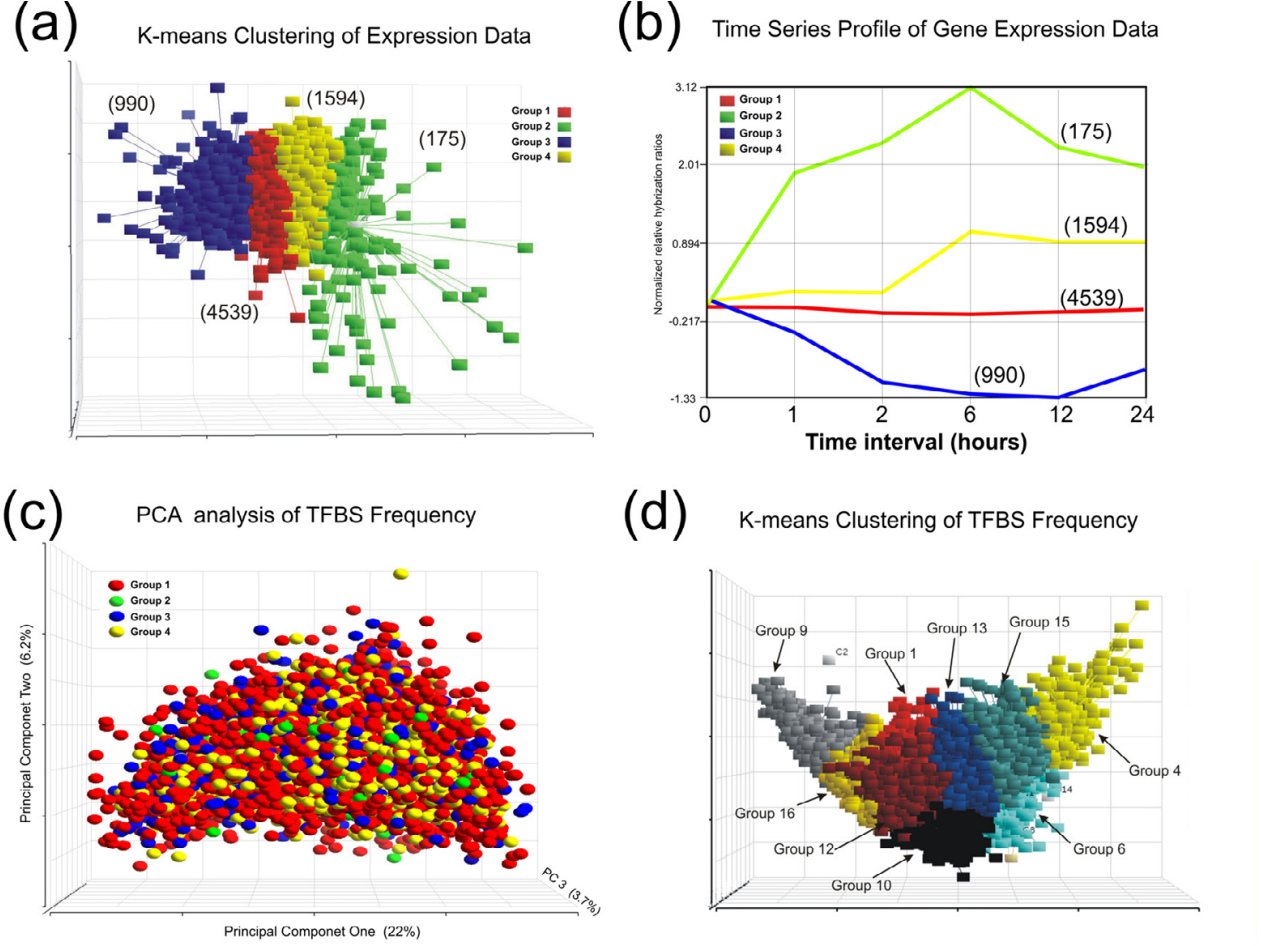
**Cluster analysis of gene expression data set from mitogen stimulated T-cells compared to promoter TFBS composition**. **(a) **K-means cluster analysis of cDNA expression profiles of phorbol ester and ionomycin stimulated Jurkat T-cells collected at 0, 1, 2, 6, 12, and 24 hours after stimulation [11]. Total genes in each cluster is indicated in parentheses. **(b) **Centroid plot representing average kinetic profiles of the four clusters at the six measured time intervals. **(c) **Principal component analysis (PCA) of TFBS frequencies in the genomic sequences extracted from the 7,298 genes profiled in Figure 1a. (1200 base pairs upstream and 200 base pairs down stream from the start of transcription). Prior to analysis, each gene was color-coded by its respective cluster shown in Figure 1a (red = cluster/group 1, no change, green = cluster/group 2 early elevated expression, blue = cluster/group 3, repressed expression, and yellow = cluster/group 4 late elevated expression). **(d) **The extracted promoter sequences of each gene were then compared with respect to TFBS composition alone by K-means clustering. Nine out of sixteen clusters contained more the 4 genes (indicated as groups 1,4,6,9,10,12,13,15, and 16).

The process of T-cell activation has been a widely applied model system for the study of stimulus-evoked transcriptional control [12]. Prior analysis of this system has shown that many of the molecular signaling pathways initiated during T-cell activation converge on RAS-dependent effectors coupled with integrated secondary messenger signaling mediated by increased calcium influx. Thus, a common means of achieving robust activation of lymphoid cell lines is through pharmacological manipulations brought about by the addition of phorbol ester and calcium ionophore to resting cells. Several recent studies have profiled time dependent changes in steady-state gene expression of mitogen-induced T-cells to search for transcriptional pathways that appeared to be disproportionately effected based on a time series analysis of the data [11,13]. The fundamental linkage between transcriptional pathways and the expressed gene is the presence of recognition motifs or TFBS within the upstream regulatory region or promoters of the pathway-influenced gene. Thus, we sought to ask whether this logic could be extended to tease out biologically significant associations between TFBS frequencies and kinetic patterns of gene expression from a previously published study of the human T-cell line Jurkat [11]. This microarray data set contains steady-state mRNA profiles measured at 0, 1, 2, 6, 12 and 24 hours following stimulation. The data set was first filtered to remove uncharacterized and poorly annotated genes (see Methods). The hybridization data from the remaining 7,298 genes was then analyzed by K-means clustering to group or classify those genes with similar kinetic patterns of mitogen-induced expression (Figure 1a). As demonstrated in Figure 1b, the expression profiles of the 7,298 genes analyzed in phorbol ester and ionomycin stimulated Jurkat T-cells can be separated into 4 kinetic clusters.

The first cluster (group 1, red) was the largest (4539) and represented genes that were essentially unchanged by mitogen stimulation. The second cluster (group 2, green) contained 175 genes and represents genes whose expression was induced early, within the first 2 hours of stimulation. The third cluster (group 3, blue) contained 990 members and represents genes that were relatively repressed by mitogen stimulation. The fourth cluster (group 4, yellow) contained 1594 members and represents genes whose expression rose late (post 6 hours) following mitogen stimulation. Given the rather broad differences between the groups and the known mitogen and calcium sensitivity of the AP-1, NF-kappa B and NFAT transcription factors pathways, it was expected that many promoters of the induced gene clusters (particular group 2) would show an asymmetric enrichment for TFBSs that bind AP-1, NF-kappa B or NFAT [12]. To address this hypothesis, 1400 bp of genomic sequence (1200 bp upstream and 200 bp downstream of the TSS) were extracted from the 7,298 genes using the ProSpector Promoter inspection tool (see Methods). These regions (referred to as promoter regions) were then scored for the presence of 164 different motifs based on the TRANSFAC 6.0 position weight matrices using the MatInspector algorithms described by Quandt et al [5]. Matrix and core thresholds were set at 0.75 and 1.0 respectively. The TFBS composition of the genes were then compared by principal component analysis (PCA), where the cluster classifications of the genes based on kinetic expression pattern were color coded (red = cluster/group 1; green = cluster/group 2, blue = cluster/group 3, and yellow = cluster/group 4). The genes were then grouped by the relative promoter frequencies of the 164 motifs applying 0.75/1.0 matrix/core PWM thresholds. In this presentation, the original 164 PWM motif vector space of the genes is reduced to 3 principal component vectors each representing a summed linear contribution from all 164 motifs [14,15]. As shown in Figure 1c, the clustering of the promoter TFBS frequencies produces a diffuse pattern that shows no correlation with the kinetic categories derived from gene expression data in Figure 1b.

These data indicate that broad kinetic grouping by gene expression alone fails to show strong correlations with transcription factor binding site composition. Though dramatic, this conclusion is not unexpected. Recent studies suggest that the correlation between steady-state mRNA levels and active transcription is at best 50%, since steady-state mRNA is the net result of not only nascent transcription, but also mRNA turnover [7]. Accordingly, promoter composition is likely to have a significantly stronger correlation with active transcription than with mRNA stability. Nonetheless, future studies aimed at generating a finer partitioning of the kinetic categories through the use of multiple conditions (e.g. different modes of stimulation) will be better prone to generate more selective gene groups with higher conditional correlation between TFBS composition and patterns of gene expression.

In clear contrast however, when the TFBS compositions of the 7,298 genes were analyzed independent of gene expression data by K-means clustering, sixteen distinct and stable clusters could be identified (Figure 1d). Seven of these clusters contained 4 or less genes and were discarded. The remaining 9 major partitions were composed of clusters containing from 271 (Cluster four) to 1266 (Cluster sixteen) genes (Figure 1d).

To determine whether there were any functional differences between the gene classes shown in Figure 1d, the genes in each cluster were analyzed for preferential enrichment or depletion of ontology terms using the GoMiner web-based software package [16]. GoMiner facilitates biological interpretation of gene lists using a quantitative statistical output that identifies gene ontology terms that are asymmetrically distributed between gene clusters. Over- and under-represented terms are ranked by a two-sided *p*-value from the Fisher's exact T-test [16]. The top 40 gene ontology terms for each gene cluster are shown in Table One. On first inspection, it is clear that each of the 9 gene clusters have distinct differences in gene ontology terms. Cluster one appears dominated by cell cycle and DNA replication terms. The immune response, defense response and cell communication terms appear to be a major discriminating feature with prominent asymmetric distribution across the gene clusters. Development, morphogenesis and differentiation terms are also major class separating terms in the gene clusters.

**Table 1:** Distribution of Ontology terms within Gene Clusters. The gene clusters identified in Figure 1d were analyzed for asymmetric distribution of ontology terms using the Gominer Software [16]. The top 40 gene ontology terms for each cluster ranked by significance scoring (Fishers exact T-test) are shown. Total numbers of genes in each cluster are indicated in parentheses. Statistical ranking of asymmetrically distributed gene ontology terms is represented by an estimated *p*-value (Fisher's Exact T-test).

	**Cluster One *(1187)***
***P-Value***	**Ontology Term**
0.0003	DNA dependent DNA replication
0.0003	mitotic cell cycle
0.0008	DNA replication
0.001	structural constituent of cytoskeleton
0.0014	metabolism
0.0015	proteolysis and peptidolysis
0.0016	cell cycle
0.0016	hydrolase activity
0.0016	S phase of mitotic cell cycle
0.0021	protein metabolism
0.0024	protein catabolism
0.0028	DNA replication and chromosome cycle
0.0029	small ribosomal subunit
0.0031	intracellular
0.0031	extracellular
0.004	DNA replication factor C complex
0.0059	nucleic acid binding activity
0.0059	ATP dependent helicase activity
0.006	transmembrane receptor protein phosphatase activity
0.006	transmembrane receptor protein tyrosine phosphatase activity
0.0061	cell proliferation
0.0063	mitochondrial inner membrane
0.0065	extracellular space
0.0065	macromolecule catabolism
0.0071	protein phosphatase activity
0.0073	nucleobase, nucleoside, nucleotide and nucleic acid metabolism
0.0074	replication fork
0.0078	protein amino acid dephosphorylation
0.0078	dephosphorylation
0.0078	protein-ligand dependent protein catabolism
0.0081	mitochondrial ribosome
0.009	inner membrane
0.0092	mitochondrion
0.0095	cellular_component unknown
0.0111	helicase activity
0.0113	organellar ribosome
0.0123	N-linked glycosylation
0.0123	di-, tri-valent inorganic cation homeostasis
0.014	proton-transporting ATP synthase complex
0.014	spindle
	
	**Cluster Nine *(724)***
***P-Value***	**Ontology Term**
0.0003	mitochondrion
0.0005	metabolism
0.0008	intracellular
0.0018	biosynthesis
0.0022	complement activation, alternative pathway
0.003	complement activation
0.0044	complement activity
0.0047	sugar binding activity
0.0047	carbohydrate binding activity
0.006	humoral defense mechanism (sensu Vertebrata)
0.0067	plasma membrane
0.007	cell adhesion molecule activity
0.0071	1-phosphatidylinositol 3-kinase complex
0.0071	membrane attack complex
0.0071	hydrolase activity, acting on acid anhydrides, catalyzing transmembrane movement of substances
0.0071	phosphatidylinositol 3-kinase activity
0.0079	ATP-binding cassette (ABC) transporter activity
0.0098	cell adhesion
0.0099	chemotaxis
0.0099	taxis
0.0125	cell-cell adhesion
0.013	mitochondrial membrane
0.0151	lectin
0.0156	G-protein coupled receptor protein signaling pathway
0.0176	cellular_component unknown
0.0187	P-P-bond-hydrolysis-driven transporter activity
0.02	thyroid hormone generation
0.02	lipid raft
0.02	ethanol oxidation
0.02	ethanol metabolism
0.02	flowering
0.02	thyroid hormone metabolism
0.02	aldo-keto reductase activity
0.02	alcohol dehydrogenase activity, iron-dependent
0.02	alcohol dehydrogenase activity, metal ion-independent
0.02	T-cell differentiation
0.02	negative regulation of Wnt receptor signaling pathway
0.02	fluid secretion
0.022	homophilic cell adhesion
0.0266	humoral immune response
	
	**Cluster Four *(271)***
***P-Value***	**Ontology Term**
0.0002	cytoplasm
0.001	transcription
0.0012	regulation of transcription, DNA-dependent
0.0013	regulation of transcription
0.0015	transcription, DNA-dependent
0.0029	immune response
0.0029	nucleus
0.0034	transferase activity, transferring sulfur-containing groups
0.0034	solute:sodium symporter activity
0.005	defense response
0.0051	phenol metabolism
0.0051	catecholamine metabolism
0.0051	organic acid transporter activity
0.0053	cell communication
0.0055	response to biotic stimulus
0.0059	protein modification
0.0063	protein kinase CK2 activity
0.0069	solute:cation symporter activity
0.0071	response to external stimulus
0.0084	negative regulation of transcription
0.0093	biogenic amine metabolism
0.0093	adherens junction
0.0096	cAMP-dependent protein kinase activity
0.0096	cyclic-nucleotide dependent protein kinase activity
0.0096	casein kinase activity
0.0097	transcription from Pol II promoter
0.0099	secretin-like receptor activity
0.0099	neurotransmitter:sodium symporter activity
0.0099	neurotransmitter transporter activity
0.0099	biogenic amine biosynthesis
0.0103	protein amino acid phosphorylation
0.0106	G-protein coupled receptor activity
0.0112	neurogenesis
0.0119	transmembrane receptor protein serine/threonine kinase signaling pathway
0.0128	phosphorylation
0.0139	small GTPase mediated signal transduction
0.0141	protein kinase activity
0.0151	brain development
0.016	frizzled receptor signaling pathway
0.016	frizzled receptor activity
	
	**Cluster Ten *(815)***
***P-Value***	**Ontology Term**
<.0001	nucleobase, nucleoside, nucleotide and nucleic acid metabolism
<.0001	nucleus
<.0001	intracellular
<.0001	extracellular space
<.0001	extracellular
<.0001	RNA binding activity
<.0001	nucleic acid binding activity
0.0001	plasma glycoprotein
0.0001	oxidoreductase activity, acting on the CH-NH2 group of donors, oxygen as acceptor
0.0003	oxidoreductase activity, acting on the CH-NH2 group of donors
0.0003	molecular_function
0.0003	alpha-type channel activity
0.0004	response to external stimulus
0.0004	channel/pore class transporter activity
0.0005	chymotrypsin activity
0.0005	RNA metabolism
0.0007	trypsin activity
0.0011	metabolism
0.0014	immune response
0.0015	defense response
0.0016	RNA processing
0.0025	response to biotic stimulus
0.0028	cell surface receptor linked signal transduction
0.0028	integral to membrane
0.0031	regulation of transcription
0.0032	transcription
0.0037	signal transducer activity
0.0039	translation regulator activity
0.004	regulation of transcription, DNA-dependent
0.004	membrane
0.0042	voltage-gated ion channel activity
0.0044	ligand-dependent nuclear receptor activity
0.0044	potassium channel activity
0.0044	steroid hormone receptor activity
0.0045	ion transport
0.005	small GTPase mediated signal transduction
0.0051	nucleoplasm
0.0052	cation channel activity
0.0054	digestion
0.0058	ligand-regulated transcription factor activity
	
	**Cluster Six *(474)***
***P-Value***	**Ontology Term**
0.0002	development
0.0002	extracellular matrix structural constituent
0.0003	muscle development
0.0004	muscle contraction
0.0007	intramolecular isomerase activity
0.0013	cell differentiation
0.0014	mitochondrion
0.002	cellular process
0.002	organogenesis
0.0022	cell adhesion
0.0027	cytoskeleton
0.0032	oncogenesis
0.0032	structural constituent of cytoskeleton
0.0033	cell communication
0.0036	morphogenesis
0.0037	troponin complex
0.0037	NGF/TNF (6 C-domain) receptor activity
0.0042	circulation
0.0046	structural molecule activity
0.0048	actin cytoskeleton
0.0049	cell motility
0.005	muscle fiber
0.0056	photoreceptor activity
0.0056	G-protein coupled photoreceptor activity
0.0056	collagen type I
0.011	intermediate filament cytoskeleton
0.011	intermediate filament
0.0125	transcription cofactor activity
0.0128	extracellular matrix structural constituent conferring tensile strength activity
0.0128	sarcomere
0.0128	myofibril
0.0128	collagen
0.0139	response to stress
0.0149	hydrolase activity
0.016	intramolecular isomerase activity, interconverting aldoses and ketoses
0.016	phosphagen metabolism
0.016	neurofilament
0.016	galactose binding lectin
0.016	inactivation of MAPK
0.0176	striated muscle thin filament
	
	**Cluster Twelve *(619)***
***P-Value***	**Ontology Term**
<.0001	cell communication
0.0001	signal transduction
0.0078	development
0.0103	phosphate metabolism
0.0103	phosphorus metabolism
0.0159	neurogenesis
0.016	cell adhesion
0.0179	intracellular signaling cascade
0.0196	amino acid transport
0.0311	small GTPase mediated signal transduction
0.0384	coreceptor activity
0.0464	heme-copper terminal oxidase activity
0.0464	acute-phase response
0.0464	regulation of metabolism
0.0476	cell-cell signaling
0.085	beta3-adrenergic receptor activity
0.085	purine ribonucleoside catabolism
0.085	purine ribonucleoside metabolism
0.085	pentose catabolism
0.085	pentose metabolism
0.085	ribose catabolism
0.085	adenosine metabolism
0.085	manganese ion transport
0.085	ADP-sugar diphosphatase activity
0.085	bile acid biosynthesis
0.0858	cellular respiration
0.094	organelle organization and biogenesis
0.0966	alcohol catabolism
0.1096	xenobiotic metabolism
0.1096	neuropeptide signaling pathway
0.1105	meiosis
0.1136	deaminase activity
0.1198	synaptic transmission
0.1215	transmission of nerve impulse
0.1314	monovalent inorganic cation transporter activity
0.1491	chloride transport
0.1627	internalization receptor activity
0.1627	regulation of mitotic cell cycle
0.1627	cAMP metabolism
0.1627	regulation of cell volume
	
	**Cluster Thirteen *(1208)***
***P-Value***	**Ontology Term**
0.0002	mitochondrion
0.0004	intracellular
0.0008	metabolism
0.0012	extracellular
0.0026	DNA repair
0.0031	immune response
0.0041	extracellular space
0.0045	phosphatidylinositol transporter activity
0.0061	cytosolic large ribosomal subunit (sensu Eukarya)
0.0065	defense response
0.0069	nucleobase, nucleoside, nucleotide and nucleic acid metabolism
0.0071	RNA binding activity
0.0078	large ribosomal subunit
0.0083	heme biosynthesis
0.0083	sex determination
0.0095	G-protein coupled receptor protein signaling pathway
0.0097	integral to membrane
0.0098	biosynthesis
0.0101	integral to plasma membrane
0.0109	mitotic cell cycle
0.0147	pigment biosynthesis
0.0147	post Golgi transport
0.015	nucleus
0.0157	cyclohydrolase activity
0.0157	protein amino acid methylation
0.0157	RNA-nucleus export
0.0157	transferase activity, transferring pentosyl groups
0.0169	porphyrin biosynthesis
0.0169	chromatin remodeling complex
0.0169	heme metabolism
0.0178	plasma membrane
0.0192	S phase of mitotic cell cycle
0.0193	coenzymes and prosthetic group biosynthesis
0.0209	cell surface receptor linked signal transduction
0.021	ion transport
0.0233	trypsin activity
0.0234	pigment metabolism
0.0236	inorganic anion transport
0.0266	apoptosis regulator activity
0.0268	nucleic acid binding activity
	
	**Cluster Fifteen *(725)***
***P-Value***	**Ontology Term**
0.0007	blood vessel development
0.0007	angiogenesis
0.001	phosphotransferase activity, alcohol group as acceptor
0.0013	nuclear localization sequence binding activity
0.0017	protein kinase activity
0.002	response to pest/pathogen/parasite
0.0023	protein serine/threonine kinase activity
0.0024	kinase activity
0.0025	cellular process
0.0028	cell migration
0.0038	actin polymerization and/or depolymerization
0.0048	spermatid development
0.0048	NLS-bearing substrate-nucleus import
0.0048	galactosyltransferase activity
0.0051	signal transduction
0.0053	protein tyrosine kinase activity
0.0059	embryogenesis and morphogenesis
0.006	neurogenesis
0.007	immune response
0.0073	cell-matrix adhesion
0.0073	nucleotide binding activity
0.0077	Golgi apparatus
0.0079	transferase activity, transferring phosphorus-containing groups
0.0088	phosphate metabolism
0.0088	phosphorus metabolism
0.0091	protein amino acid phosphorylation
0.0097	response to wounding
0.0097	response to biotic stimulus
0.0106	phosphorylation
0.0111	RAN protein binding activity
0.0112	morphogenesis
0.0113	development
0.0113	purine nucleotide binding activity
0.012	actin filament-based process
0.0121	importin, beta-subunit
0.0121	actin modulating activity
0.0121	actin monomer binding activity
0.0121	regulation of actin polymerization and/or depolymerization
0.0124	cytoskeleton organization and biogenesis
0.0137	cell communication
	
	**Cluster Sixteen *(1266)***
***P-Value***	**Ontology Term**
0.0004	immune response
0.0008	oncogenesis
0.0009	defense response
0.0042	ionic insulation of neurons by glial cells
0.0125	inflammatory response
0.0245	histogenesis and organogenesis
0.0261	sarcomere alignment
0.0261	phagocytosis, engulfment
0.0261	negative regulation of osteoclast differentiation
0.0261	regulation of osteoclast differentiation
0.0261	negative regulation of cell differentiation
0.0261	NO mediated signal transduction
0.0326	activation of NF-kappaB-inducing kinase
0.0327	oogenesis
0.0453	cell activation
0.0483	humoral immune response
0.0491	protein modification
0.0575	regulation of cell differentiation
0.0673	cell cycle
0.07	biotin metabolism
0.0806	phosphate metabolism
0.0806	phosphorus metabolism
0.0888	sensory organ development
0.0888	G-protein signaling, adenylate cyclase activating pathway
0.1073	pattern specification
0.1111	gametogenesis
0.119	peptide receptor activity
0.1221	microtubule-based process
0.1251	phosphate transport
0.1251	glutathione conjugation reaction
0.1251	G-protein chemoattractant receptor activity
0.1256	phagocytosis
0.1256	carbohydrate kinase activity
0.1299	regulation of transcription
0.1309	fatty acid metabolism
0.1435	antimicrobial humoral response (sensu Invertebrata)
0.1435	protein amino acid phosphorylation
0.1454	NIK-I-kappaB/NF-kappaB cascade
0.1507	protein phosphatase type 2C activity
0.1507	heavy metal ion transport

**Figure 2:**
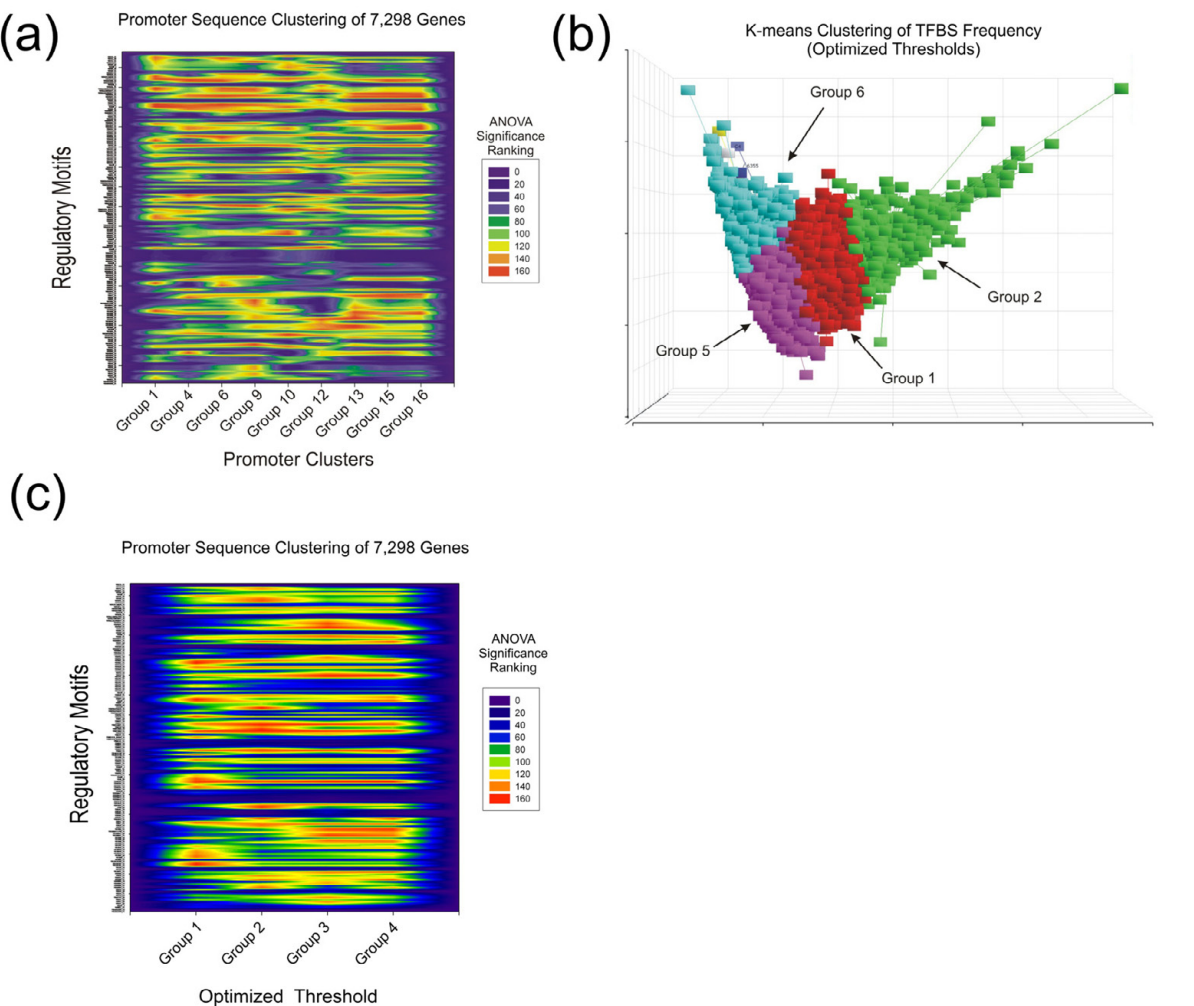
**Significance ranking of TFBSs in respective clusters**. (**a**) The TFBSs in the sequences of each cluster were sorted and ranked by ANOVA analysis to determine those sites that best discriminated the different clusters. The TFBSs in each cluster were then assigned ranks (1–164) according to their significance (*p*-value) from the ANOVA analysis. Highest ranking in red, lowest in blue. (**b**) Partitioning of gene promoter composition with more stringent PWM matrix similarity thresholds reduces the number of clusters identified by K-means analysis. Shown are four of six clusters containing greater than 4 genes. (groups 1, 2, 5 and 6). (**c**) Analysis of the most discriminating TFBSs in the four clusters in Figure 2b by ANOVA, as in Figure 2a.

To determine which TFBSs were most important for discriminating the different gene clusters, the 164 motifs were ranked for significance in each cluster by ANOVA assigned significance based on discriminatory power. The significance ranking was derived from the *p*-value output for each motif in each cluster and then converted to a color score based on the ranking (1–164 = low-high = blue-red). A contour heat diagram showing the differential ranking of the 164 motifs in each of the 9 clusters is shown in Figure 2a. As apparent from this heat diagram, the TFBS patterns of the majority of the clusters produce very distinct signatures (Figure 2a).

When the TFBS composition of the 7,298 promoter regions was scored using more stringent PWM thresholds optimized to yield fewer potential false positive predictions (Methods), a surprising decrease in the diversity of the clustering pattern for the promoters was observed (Figure 2b). The analysis predicted 6 clusters from which two were discarded for having fewer than 4 genes. As expected, a heat diagram of the 164 TFBS rankings in each of the clusters showed considerably less distinct signatures (Figure 2c). Moreover, the ontology terms associated with the 4 clusters were much less distinct with a higher total ratio of redundant terms (see supplemental data, Table two). This tendency for more relaxed PWM similarity thresholds to generate greater diversity in predicted promoter composition suggests that the inherent or "perceived" degenerate nature of transcription factor binding sites serves to broaden the potential "categories" or strategies of gene regulation [17]. This suggests high thresholds, though reducing the number of potential false positives, have the severe negative effect of overlooking real binding sites [17].

**Figure 3:**
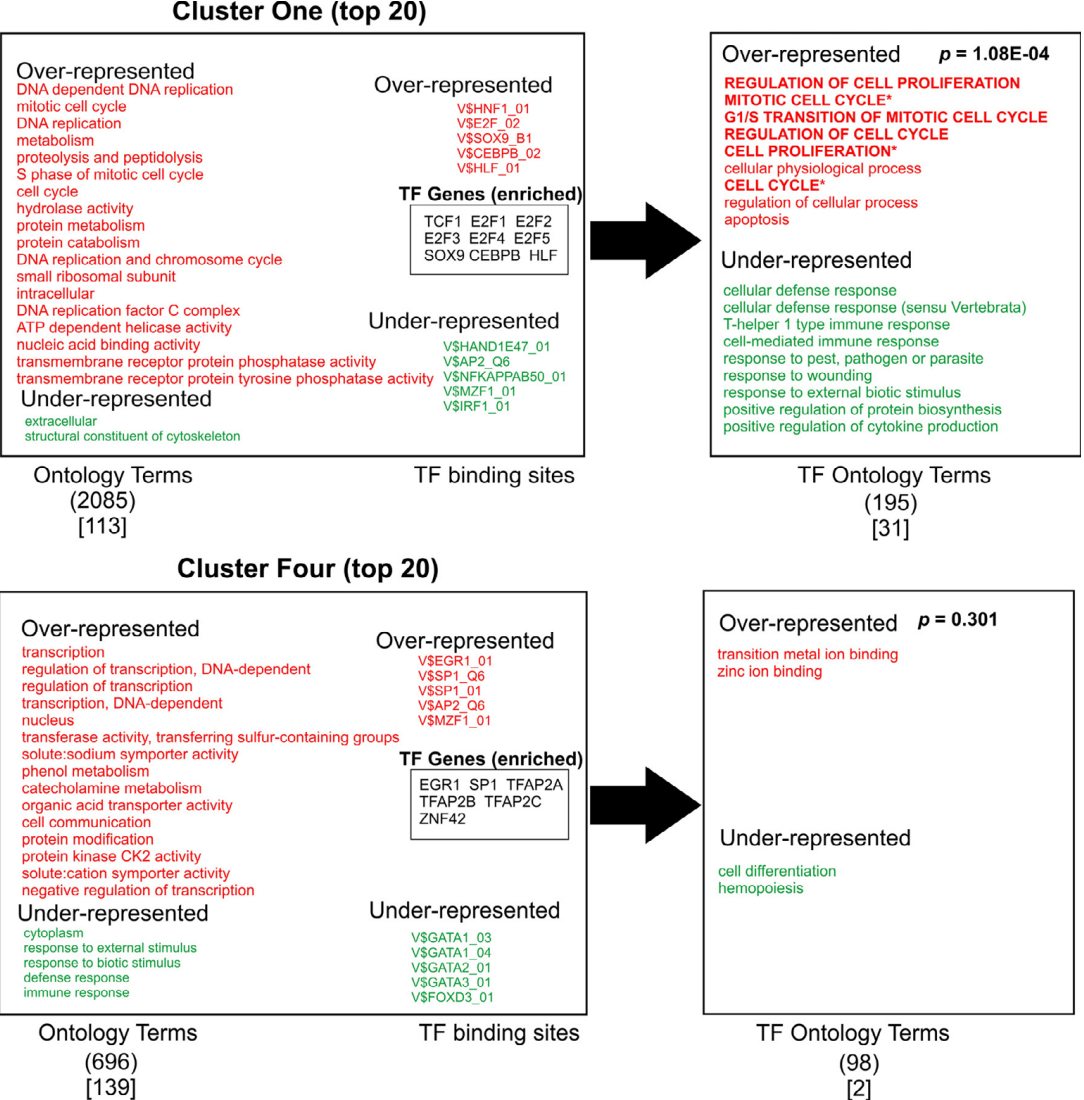
**Analysis of Ontology term distribution**. The top 20 best discriminating gene ontology terms in each cluster were sorted for over-representation (RED) and under-representation (Green) and compared to the top 10 discriminating TFBSs for each cluster as determined by ANOVA (Figure 2). The top 10 over-represented (Red) and under-represented (Green) TFBSs for each cluster are shown. The transcription factors that recognize the TFBSs were grouped and then analyzed for asymmetric distribution of ontology terms using GoMiner (TF ontology terms, right). Transcription factor genes that are known to bind the over-represented TFBSs (TF Genes, enriched) are shown enclosed in boxes. Transcription factor ontology terms that overlap the gene cluster ontology terms within 2 branches of the ontology clade are shown in bold. Those terms with exact matches in the gene cluster ontologies are indicated with an asterisk. The numbers in parentheses indicate the total number of ontology terms associated with each respective cluster. The numbers in brackets indicated those ontology terms with a significance measurement *p*-value < 0.05 (Fisher Exact T-test). Representative genes from Clusters one, six and thirteen are shown in supplemental Table 3.

**Figure 4:**
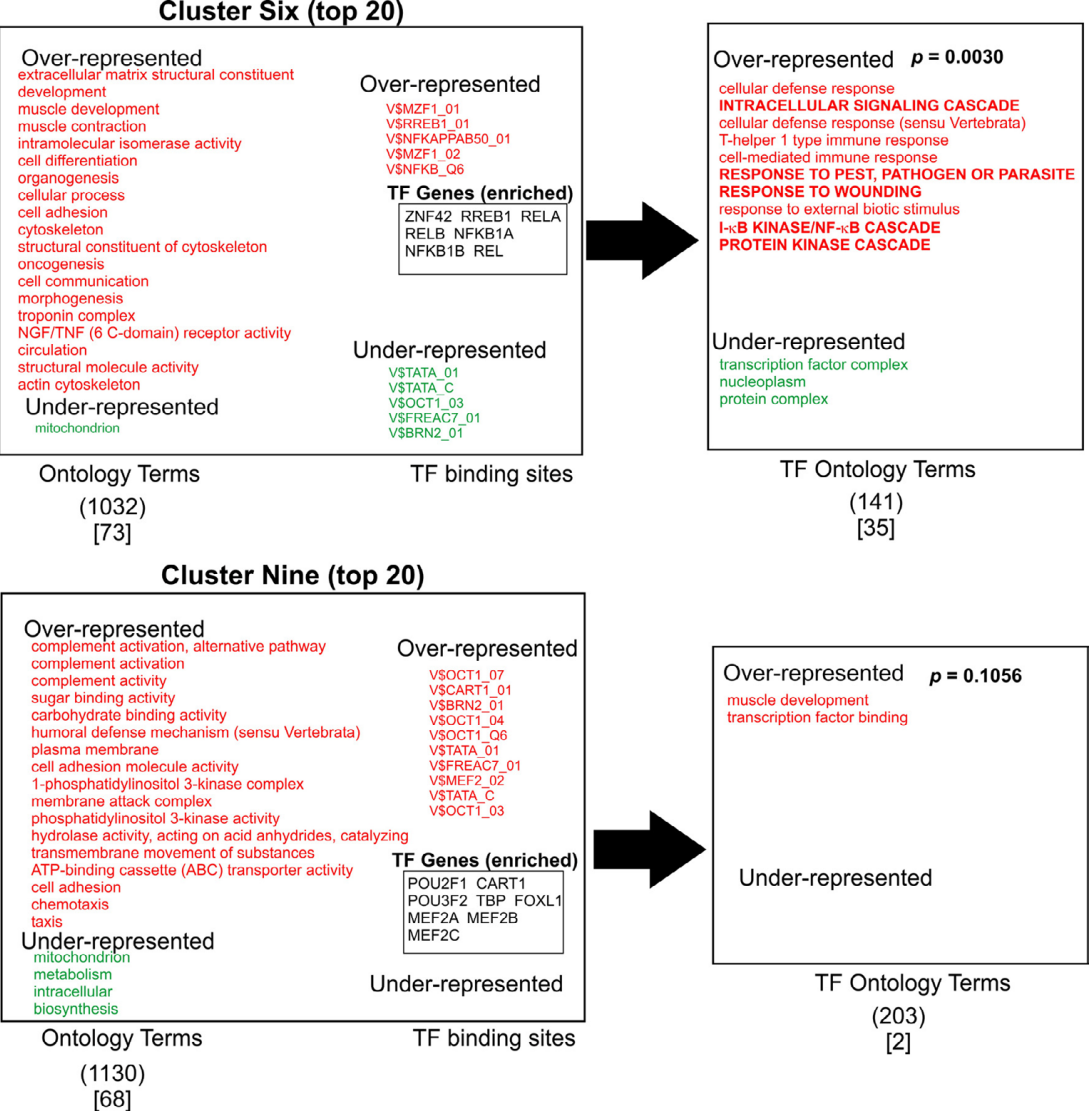
**Analysis of Ontology term distribution**. The top 20 best discriminating gene ontology terms in each cluster were sorted for over-representation (RED) and under-representation (Green) and compared to the top 10 discriminating TFBSs for each cluster as determined by ANOVA (Figure 2). The top 10 over-represented (Red) and under-represented (Green) TFBSs for each cluster are shown. The transcription factors that recognize the TFBSs were grouped and then analyzed for asymmetric distribution of ontology terms using GoMiner (TF ontology terms, right). Transcription factor genes that are known to bind the over-represented TFBSs (TF Genes, enriched) are shown enclosed in boxes. Transcription factor ontology terms that overlap the gene cluster ontology terms within 2 branches of the ontology clade are shown in bold. Those terms with exact matches in the gene cluster ontologies are indicated with an asterisk. The numbers in parentheses indicate the total number of ontology terms associated with each respective cluster. The numbers in brackets indicated those ontology terms with a significance measurement *p*-value < 0.05 (Fisher Exact T-test). Representative genes from Clusters one, six and thirteen are shown in supplemental Table 3.

**Figure 5:**
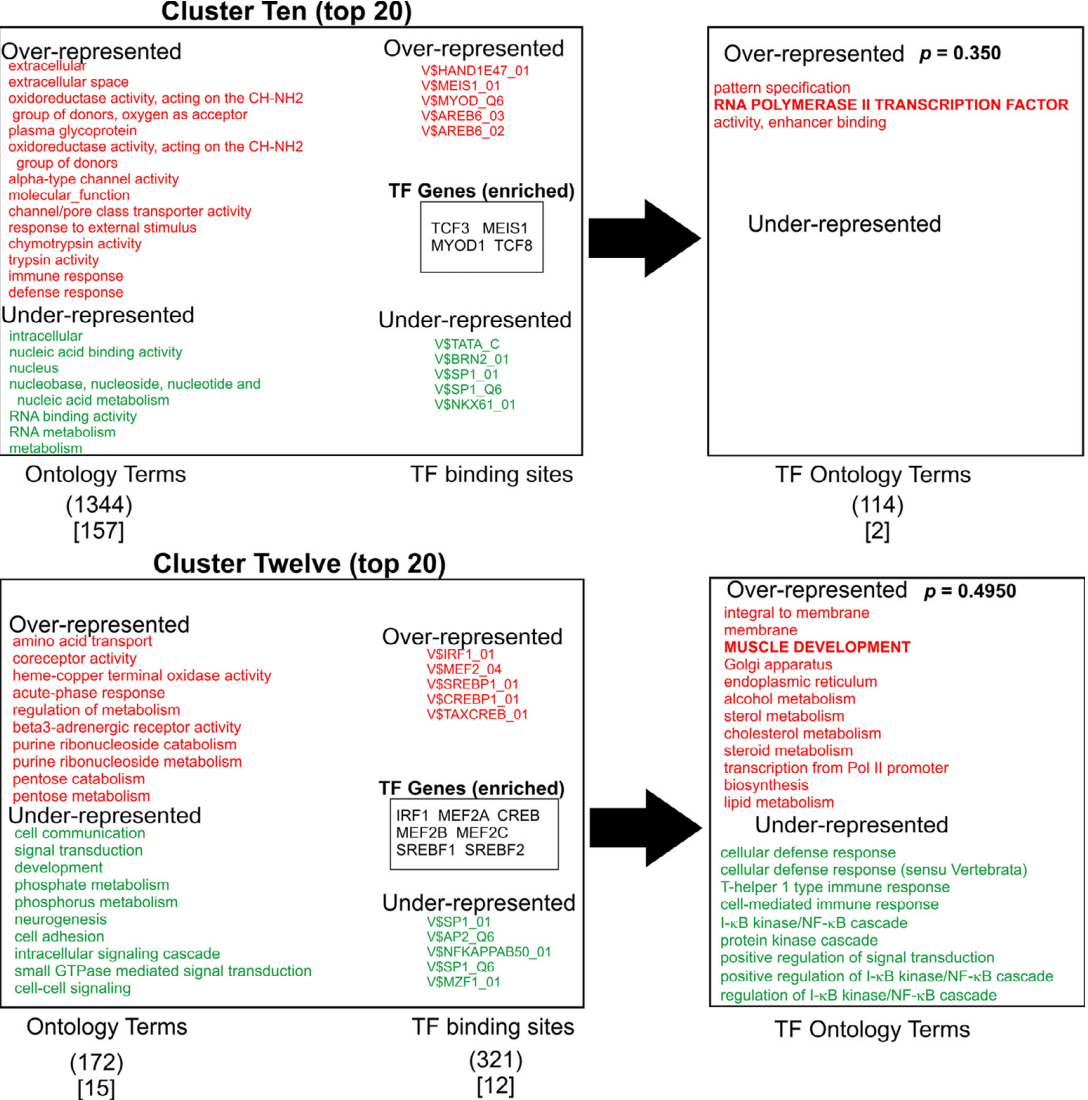
**Analysis of Ontology term distribution**. The top 20 best discriminating gene ontology terms in each cluster were sorted for over-representation (RED) and under-representation (Green) and compared to the top 10 discriminating TFBSs for each cluster as determined by ANOVA (Figure 2). The top 10 over-represented (Red) and under-represented (Green) TFBSs for each cluster are shown. The transcription factors that recognize the TFBSs were grouped and then analyzed for asymmetric distribution of ontology terms using GoMiner (TF ontology terms, right). Transcription factor genes that are known to bind the over-represented TFBSs (TF Genes, enriched) are shown enclosed in boxes. Transcription factor ontology terms that overlap the gene cluster ontology terms within 2 branches of the ontology clade are shown in bold. Those terms with exact matches in the gene cluster ontologies are indicated with an asterisk. The numbers in parentheses indicate the total number of ontology terms associated with each respective cluster. The numbers in brackets indicated those ontology terms with a significance measurement *p*-value < 0.05 (Fisher Exact T-test). Representative genes from Clusters one, six and thirteen are shown in supplemental Table 3.

**Figure 6:**
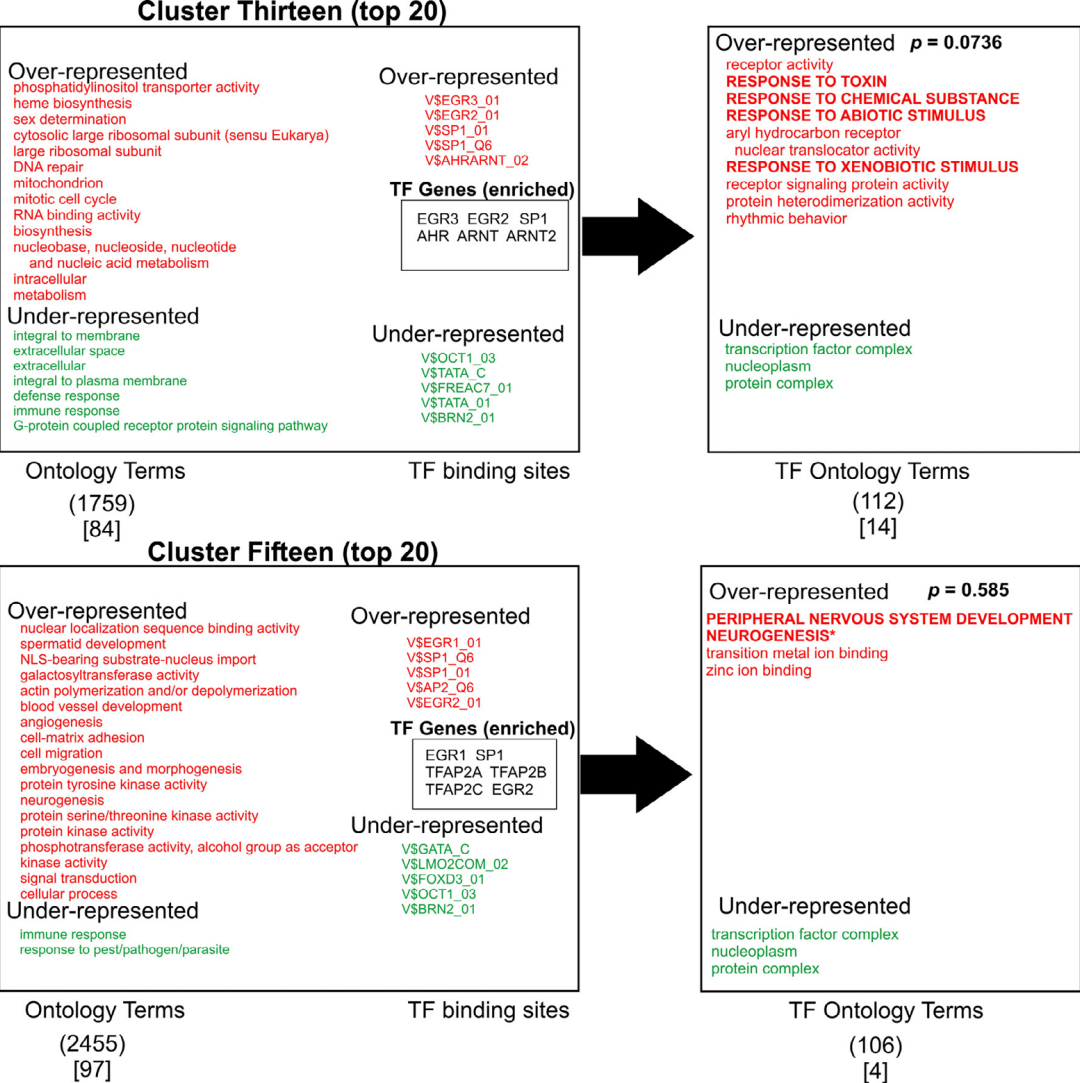
**Analysis of Ontology term distribution**. The top 20 best discriminating gene ontology terms in each cluster were sorted for over-representation (RED) and under-representation (Green) and compared to the top 10 discriminating TFBSs for each cluster as determined by ANOVA (Figure 2). The top 10 over-represented (Red) and under-represented (Green) TFBSs for each cluster are shown. The transcription factors that recognize the TFBSs were grouped and then analyzed for asymmetric distribution of ontology terms using GoMiner (TF ontology terms, right). Transcription factor genes that are known to bind the over-represented TFBSs (TF Genes, enriched) are shown enclosed in boxes. Transcription factor ontology terms that overlap the gene cluster ontology terms within 2 branches of the ontology clade are shown in bold. Those terms with exact matches in the gene cluster ontologies are indicated with an asterisk. The numbers in parentheses indicate the total number of ontology terms associated with each respective cluster. The numbers in brackets indicated those ontology terms with a significance measurement *p*-value < 0.05 (Fisher Exact T-test). Representative genes from Clusters one, six and thirteen are shown in supplemental Table 3.

**Figure 7:**
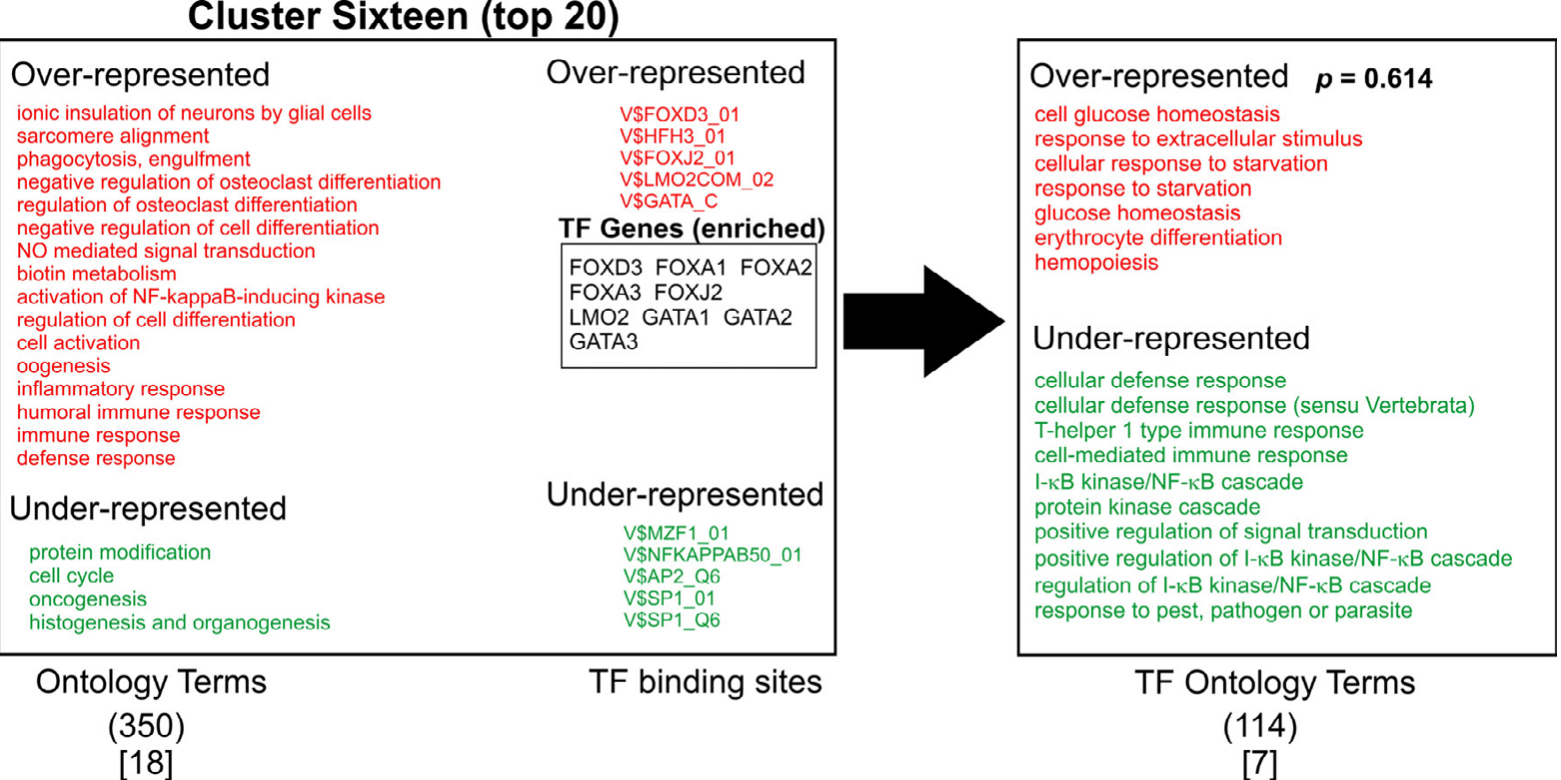
**Analysis of Ontology term distribution**. The top 20 best discriminating gene ontology terms in each cluster were sorted for over-representation (RED) and under-representation (Green) and compared to the top 10 discriminating TFBSs for each cluster as determined by ANOVA (Figure 2). The top 10 over-represented (Red) and under-represented (Green) TFBSs for each cluster are shown. The transcription factors that recognize the TFBSs were grouped and then analyzed for asymmetric distribution of ontology terms using GoMiner (TF ontology terms, right). Transcription factor genes that are known to bind the over-represented TFBSs (TF Genes, enriched) are shown enclosed in boxes. Transcription factor ontology terms that overlap the gene cluster ontology terms within 2 branches of the ontology clade are shown in bold. Those terms with exact matches in the gene cluster ontologies are indicated with an asterisk. The numbers in parentheses indicate the total number of ontology terms associated with each respective cluster. The numbers in brackets indicated those ontology terms with a significance measurement *p*-value < 0.05 (Fisher Exact T-test). Representative genes from Clusters one, six and thirteen are shown in supplemental Table 3.

A logical prediction in this study is that the associated biological function of the transcription factors that regulate the gene groups should share some similarity with the function of the genes that they regulate. In other words, the "regulator" should show similar function to the "regulated". To ask this question, we looked for any correlation between the ontology terms of the transcription factors (TFs) predicted most likely to bind to the promoter regions of the gene clusters (TFO) and the ontology terms of the gene clusters themselves (GCO). Accordingly, a list of transcription factors known to recognize the most discriminating TFBSs for each cluster was generated (total of 55 TF genes, Figures 3, 4, 5, 6, 7). The top 10 TFBSs were segregated into over-represented (RED) or under-represented (GREEN) groups for their respective gene clusters. The list of genes encoding the transcription factors that bind the top 10 TFBSs in each cluster was then compiled based on TRANSFAC 6.0 annotation. The GoMiner software was then used to rank the gene ontology terms based on the statistical significance of their occurrence within the transcription factor clusters (TFO). The top gene ontology terms with a ranking *p*-value less than 0.05 (determined by GoMiner) were then extracted and listed depending on whether they were over-represented (RED) or under-represented (GREEN) in each TFO. These lists were then compared with the top gene ontology terms of each respective gene cluster (gene cluster ontology, GCO) with *p*-values less then 0.05. The over-represented transcription factor ontology terms (TFO) found to share similarity with terms in the respective gene cluster ontologies (GCO) (within two branches of the ontology clade) are displayed in bold capitals letters (Figures 3, 4, 5, 6, 7, right column).

A qualitative comparison of the gene cluster ontology terms and their respective transcription factor ontology terms reveals several similarities in the over-represented terms. Cluster one shows significant overlap of ontology terms for cell division. Cluster six shows overlapping terms for cell communication. Cluster twelve contained overlapping terms for cellular metabolism. Cluster thirteen shows a puzzling anti-correlation with response to external stimuli. Cluster fifteen shows overlapping terms with morphogenesis and development. When these correlations are tested for significance by the method of hyper-geometric distribution, Clusters one and six shows statistically significant correlation. Within Cluster one, gene cluster and transcription factor ontology terms for cell cycle regulation overlapped significantly (*p *= 1.08E-04). Within Cluster six, there was substantial overlap for cell communication ontology terms (*p *= 0.0030). Both cell cycle regulation and cell communication encompass fundamental and highly conserved processes in mammalian cells. Less than a third of clusters showed statistically significant correlations between gene group and transcription factor ontology terms. Nonetheless, given the unbiased manner in the which the gene lists and TF lists were generated and the small number of TF genes used to generate that TFO terms (55) compared to the number used to generate the GCO terms (7298), this approach shows substantial promise for identifying functional correlations between the transcriptional pathways and the genes regulated by them. It is reasonable to anticipate that these correlations will strengthen as the number and quality of the PWMs expand and the transcription factor gene ontology annotation improves in number and accuracy (see Discussion).

## Discussion

Changes or alteration in gene expression are often linked to influences at the regulatory elements within the promoter regions of the targeted genes. The transcription factors that bind these regulatory elements form the final controlling functional link to the signaling pathways that are triggered and integrated as the cell adapts to environmental change. Thus, collective control of these integrated pathways forms the major conduit that governs changes and patterns of cellular behavior. These relationships are particularly applicable to metazoan systems.

Transcriptional control in metazoan cells is the culmination of multiple signal-induced transcriptional pathways, where the collective influence of more than one transcription factor and pathway hold sway on the ultimate expression of targeted genes. This combinatorial logic provides a means through which a finite number of transcriptional pathways can converge to produce seemingly infinite patterns of gene regulatory control. Deciphering this logic and how it links downstream function to upstream signaling requires expanded methods of interpreting promoter composition. By classifying patterns of promoter composition and linking these classifications to functional categories, of both the regulated genes and the transcription factors that regulate them, this approach provides a rational method for identifying meaningful relationships between promoter composition and gene function.

Though only 2 of 9 clusters showed a statistically significant correlation between ontology terms of the clusters and the transcription factors (Figures 3, 4, 5, 6, 7), an inspection of the ontology terms of several of the gene clusters in comparison to the transcription factors reveals numerous relationships that have been well established in the literature, though not reflected in the currently available ontological annotation for the factors. Cluster one is dominated by E2F transcription factors that are well known to exert control over genes involved in cell cycle regulation. Therefore, the overlap between Cluster one gene and transcription factor ontology terms for cell cycle regulation are significant (*p*-value = 1.08E-04). Cluster Four showed no matches in the most significant ontology terms, however, the significant potential regulators of this cluster include AP-2 transcription factors, which have broad roles in vertebrate development including control of apoptosis and cell cycle [18]. Moreover, AP-2 factors have been also found to control receptor tyrosine kinase expression and other factors involved in the negative regulation of gene expression [19,20]. EGR1 and ZNF42 factors are widely known to regulate genes important for mitogenesis and differentiation [21-23]. Thus, a more expanded annotation of these terms would have shown greater correlation with the top ontology terms in both Cluster four and Cluster fifteen. These include cell communication, negative regulation of transcription, protein modification, protein tyrosine kinase activity, embryogenesis, morphogenesis, signal transduction and angiogenesis (Figures 3 and 6). Even though Cluster six shows statistically significant overlap between its ontology terms and those of its potential regulating transcription factors, many seemingly obvious matches could not be found in the annotation of some of the potential regulators. In particular, there is a significant absence of ontology terms for oncogenesis, morphogenesis and cellular differentiation for the NF-kappa B family subset of the top discriminating transcription factors. Control of these cellular process are well described for NF-kappa B [24]. The transcription factors in Cluster nine show no overlap; yet, it is dominated by octamer binding sites and several reports indicate octamer family members have a role in the control of expression of cellular adhesion molecules and other participants in wound healing [25,26]. In Cluster ten, the roles for TCF3 and TCF8 in early B-cell differentiation, immunoglobin expression and T-cell function certainly should have produced an overlap with the gene cluster ontology terms for immune response and defense response [27-29].

Another dominant factor that will improve the deduced linkages between ontologies of the regulating transcription factors and the regulated genes will be improvements in the accuracy of predicting TFBS occurrence. Multiple difficult factors have to be addressed. The first is accurate prediction of the promoter regions themselves. In this work, we define the promoter region in terms of the start of transcription (TSS) and retrieve sequence 200 bp downstream and 1200 bp upstream of this position. Using the ProSpector search engine, the TSS is extracted from RefSeq annotation provided by the USCS genome assembly [30]. More precise identification of TSS is available from recent curated databases containing empirically derived TSS positions such as MPromDb , OMGProm , and DBTSS [31,32]. These resources will certainly improve on the accuracy of the promoter identification as their inventories continue to grow from the current 8,793 (DBTSS) and 13,780 (MPromDb) human genes. Nonetheless, a comparison of the promoter sequences queried from ProSpector and those from MPromDb showed a greater than 80% overlap in more than 80% of the mutually retrieved sequences (data not shown). It should be noted that metazoan promoter regions are highly complex and have multiple different TSS positions and consequently multiple promoters [33]. Many of these alternate promoters are tissue specific [33]. This feature unavoidably confounds the approach and is not adequately addressed in current promoter analysis tools. In addition to alternate promoters, metazoan gene regulatory regions are influenced by distant enhancer regions, locus control regions and a complicated tissue-specific interplay between transcriptional co-activator complexes and transcription factors [1,34-36]. These complex factors probably account for the better performance of promoter prediction tools on yeast data sets in comparison to higher eukaryotes [37].

A particularly difficult problem with the use of PWMs to annotate gene regulatory regions is the unavoidable occurrence of "false positive" and "false negatives". This is predominantly the case when searching for new TFBSs in uncharacterized genetic regions. Figures 2b and 2c show that using a high PWM threshold has the negative result of reduced promoter discrimination and potentially high levels of true "false negatives". At the heart of the matter is how we discriminate true false negatives and positives. It is indisputable that this can only be done through empirical validation and verification. The thresholds set for the PWM analysis in figures 2b and 2c were too high to detect known sites for CREB, AP1, NF-kappa B and NFAT in the IL2 promoter and failed to retrieve any of the 5 known sites for NFAT in the IL4 promoter [38-43]. Thus, the use of high thresholds is inappropriate. For empirically uncharacterized gene regulatory regions, there is no way to discriminate between correct identifications, true false positives or false negatives. High frequency occurrence of motifs in some promoters should be met with some skepticism, but it is important to keep in mind that our understanding of transcription factor interaction with the genome continues to evolve. The interaction between transcription factors and TFBSs is not static, but highly dynamic and repetitive [44]. Thus, gradients of high and low affinity binding sites for classes of factors within a single gene regulatory locus could be physiologically relevant.

The presence of GC rich regions and CpG islands creates an important issue that requires consideration. These types of regions contain high densities of binding sites for factors such as Sp1, AP2, and EGR2/3. Though greater than 80% of promoters of are thought to contain CpG islands [45,46], differences in their presence, length or position will lead to background noise in the analysis. Recently developed approaches are able to address this problem through the use of background models representing either the entire genome (which is still subject to GC rich asymmetry because of the preferential concentration at transcription start sites) or random/unselected groups of promoter regions [47,48]. The method described in this current study ranks motifs not by their PWM score, but by using ANOVA to discriminate across the opposing clusters. By this approach, the aggregate of the opposing clusters serves as the background model for discriminatory significance of the TFBSs within each group. Though the presence of GC rich regions contribute significant noise to the analysis, this problem does not overwhelm the approach since it robustly discriminates true differences in promoter composition and correctly groups genes of known ontology with those containing mutual TFBSs that have been empirically validated (see supplemental Table 3).

Only Clusters fifteen, thirteen and four show high ranks for GC-containing TFBSs (Figures 3 and 6). At the very least, this indicates that there is a non-random distribution of GC rich regions amongst promoters. Nonetheless, the relative contribution of GC rich tracts or CpG islands to this distribution cannot be determined by our method. As expected, the clusters with high ranks for GC-containing TFBSs are in close apposition (Figure 1d). The fact that they show rather low correlation between GCO and TFO reflects the noise due to the high occurrence of GC rich regions. Still, it must be emphasized that CpG islands represent legitimate sites for factors like Sp1, EGR3/2 and AP2. Thus, the detection of such binding sites is likely to be physiologically important and their clustering patterns may contain biological information that will increase in importance as our understanding of transcription factor ontology is refined. Interestingly, recent studies suggest that genes lacking CpG islands tend to be expressed with a higher degree of tissue specificity and contain more GO terms consistent with "signal transducer", confirming the speculation that many CpG islands are associated with house-keeping gene function [46].

Unlike the yeast studies of Tavazoie et al [49], our approach failed to show any correlation between gene expression patterns at the RNA level and promoter TFBS composition. This result could be due in large part to the differences in yeast and metazoan gene regulatory regions as discussed above. In addition, post-transcriptional regulation of RNA stability is likely to be much more complex in metazoans than yeast. Another very important consideration is that Tavazoie et al chose to study the cell cycle, a time series of cellular behavior that is rich in various distinct molecular programs. It may be that the use of time-dependent changes following mitogen stimulation is too broad and lacks sufficient variability and distinction of gene expression to provide the discriminatory power necessary for the analysis of gene regulatory regions. Recently, another group has made an elegant application of GO terms to predict biological function by promoter composition [50]. By this approach, Bluthgen et al used predetermined TFBS combinations of known biological significance to extract genes with similar biological function based on overlapping promoter composition. This approach is very promising, confirms our central hypothesis, and shares similarity with a previously reported method where the biological significance of TFBS combinations, derived from kinetic profiles of transcriptional regulator occupancy via chromatin immuno-precipitation, was used to identify similarly regulated genes [14]. Our analysis differs from Bluthgen et al in that it does not depend on prior knowledge. In contrast, it begins with neither a pre-selected TFBS framework nor any other biological information. The possibility of identifying previously unrecognized ontologies linking the targeted genes with their targeting transcription factors remains preserved.

## Conclusion

The examination of the upstream regulatory sequences of eukaryotic genes has the potential of yielding a wealth of information that will unravel the transcriptional control codes that govern spatial and temporal changes in gene expression. Combining multivariate analysis of promoter composition with classification by gene ontology provides a method that defines functional links between regulated genes and the genes that regulate them. The above are just a few of the examples where expanded ontology term annotation for the transcription factors based on current literature and improved promoter annotation methods will enhance the functional correlation between the regulator and the regulated. Just as important, these examples also point out how this method may also aid in identifying previously unrecognized functions for known transcription factors through the identification of "mutual ontology" terms. Ultimately, it will be the broader refinement and expansion of both PWMs for TFBSs and the functional vocabulary for all genes (in particular those gene encoding transcription factors), that will have a significant impact on improving the utility of this approach.

## Methods

### ProSpector database

To aid in the extraction and analysis of human promoter regions, a web-based resource named ProSpector (PROmoter inSPECTion) was developed [51]. The ProSpector website operates through an Apache web server and a MySQL relational database. The user interface of the website is written in PHP. The website provides a search tool for retrieving oriented human gene promoter regions by gene name (HUGO), gene description, gene symbol (HUGO), UniGene cluster, RefSeq ID, or LocusLink ID. The search feature is facilitated by keeping local copies of NCBI databases, including UniGene, RefSeq, and LocusLink. The actual promoter sequences are retrieved through a tool developed by the Genome Analysis Unit of the National Cancer Institute [52]. This tool retrieves promoters by extracting regions 5' of gene transcription start sites identified by RefSeq annotation. Transcription start is defined by RefSeq mRNAs aligned with genomic chromosomal contigs from the UCSC/NCBI Assembly – hg12/Build 30. ProSpector also allows extracted promoters to be analyzed for putative transcription factor binding sites using the MatInspector algorithms described by Quandt et al [5] and a subset of the position weight matrices (PWM) from the TRANSFAC 6.0 public database of transcription factors [53]. Briefly, base composition at putative TFBS is calculated as a vector score (C_*i*_(*i*)) where:

Ci(i)=(100/ln5)×[∑b∈A,C,G,T,gap[P(i,b)×lnP(i,b)]+ln5]     (1)
 MathType@MTEF@5@5@+=feaafiart1ev1aaatCvAUfKttLearuWrP9MDH5MBPbIqV92AaeXatLxBI9gBaebbnrfifHhDYfgasaacH8akY=wiFfYdH8Gipec8Eeeu0xXdbba9frFj0=OqFfea0dXdd9vqai=hGuQ8kuc9pgc9s8qqaq=dirpe0xb9q8qiLsFr0=vr0=vr0dc8meaabaqaciGacaGaaeqabaqabeGadaaakeaacqWGdbWqdaWgaaWcbaGaemyAaKgabeaakiabcIcaOiabdMgaPjabcMcaPiabg2da9iabcIcaOiabigdaXiabicdaWiabicdaWiabc+caVmXvP5wqSXMqHnxAJn0BKvguHDwzZbqegyvzYrwyUfgaiqaacaWFSbGaeiOBa4MaeGynauJaeiykaKIaey41aq7aamWaaeaadaaeqbqaamaadmaabaGaemiuaaLaeiikaGIaemyAaKMaeiilaWIaemOyaiMaeiykaKIaey41aqRaa8hBaiabc6gaUjabdcfaqjabcIcaOiabdMgaPjabcYcaSiabdkgaIjabcMcaPaGaay5waiaaw2faaaWcbaGaemOyaiMaeyicI4SaemyqaeKaeiilaWIaem4qamKaeiilaWIaem4raCKaeiilaWIaemivaqLaeiilaWIaem4zaCMaemyyaeMaemiCaahabeqdcqGHris5aOGaey4kaSIaeeiBaWMaeiOBa4MaeGynaudacaGLBbGaayzxaaGaaCzcaiaaxMaadaqadaqaaiabigdaXaGaayjkaiaawMcaaaaa@7707@

P(*i*, b) being the relative frequency of base (*b*) at position (*i*) calculated from the PWM. Core similarity is used to quickly screen for potential binding sites with a high similarity to the most conserved region of the PWM and is determined from the four consecutive bases in the PWM with the highest Ci and calculated using:

core_sim=[∑j=mm+3score(b,j)]/[∑j=mm+3max_score(j)]     (2)0≤core_sim≤1
 MathType@MTEF@5@5@+=feaafiart1ev1aaatCvAUfKttLearuWrP9MDH5MBPbIqV92AaeXatLxBI9gBaebbnrfifHhDYfgasaacH8akY=wiFfYdH8Gipec8Eeeu0xXdbba9frFj0=OqFfea0dXdd9vqai=hGuQ8kuc9pgc9s8qqaq=dirpe0xb9q8qiLsFr0=vr0=vr0dc8meaabaqaciGacaGaaeqabaqabeGadaaakqaabeqaaiabdogaJjabd+gaVjabdkhaYjabdwgaLjabc+faFjabdohaZjabdMgaPjabd2gaTjabg2da9maadmaabaWaaabCaeaacqWGZbWCcqWGJbWycqWGVbWBcqWGYbGCcqWGLbqzcqGGOaakcqWGIbGycqGGSaalcqWGQbGAcqGGPaqkaSqaaiabdQgaQjabg2da9iabd2gaTbqaaiabd2gaTjabgUcaRiabiodaZaqdcqGHris5aaGccaGLBbGaayzxaaGaei4la8YaamWaaeaadaaeWbqaaiabd2gaTjabdggaHjabdIha4jabd+faFjabdohaZjabdogaJjabd+gaVjabdkhaYjabdwgaLjabcIcaOiabdQgaQjabcMcaPaWcbaGaemOAaOMaeyypa0JaemyBa0gabaGaemyBa0Maey4kaSIaeG4mamdaniabggHiLdaakiaawUfacaGLDbaacaWLjaGaaCzcamaabmaabaGaeGOmaidacaGLOaGaayzkaaaabaGaeGimaaJaeyizImQaem4yamMaem4Ba8MaemOCaiNaemyzauMaei4xa8Laem4CamNaemyAaKMaemyBa0MaeyizImQaeGymaedaaaa@7F77@

The score for base (*b*) and position (*j*) is simply the matrix value of base (*b*) at position (*j*) as defined by the PWM and the max score is the highest value in the matrix at position (*j*). Likeness or similarity to the TFBS PWM is calculated independent of the core by:

mat_sim=[∑j=1nCi(j)×score(b,j)]/[∑j=1nCi(j)×max_score(j)]     (3)0≤mat_sim≤1
 MathType@MTEF@5@5@+=feaafiart1ev1aaatCvAUfKttLearuWrP9MDH5MBPbIqV92AaeXatLxBI9gBaebbnrfifHhDYfgasaacH8akY=wiFfYdH8Gipec8Eeeu0xXdbba9frFj0=OqFfea0dXdd9vqai=hGuQ8kuc9pgc9s8qqaq=dirpe0xb9q8qiLsFr0=vr0=vr0dc8meaabaqaciGacaGaaeqabaqabeGadaaakqaabeqaaiabd2gaTjabdggaHjabdsha0jabc+faFjabdohaZjabdMgaPjabd2gaTjabg2da9maadmaabaWaaabCaeaacqWGdbWqdaWgaaWcbaGaemyAaKgabeaakiabcIcaOiabdQgaQjabcMcaPiabgEna0kabdohaZjabdogaJjabd+gaVjabdkhaYjabdwgaLjabcIcaOiabdkgaIjabcYcaSiabdQgaQjabcMcaPaWcbaGaemOAaOMaeyypa0JaeGymaedabaGaemOBa4ganiabggHiLdaakiaawUfacaGLDbaacqGGVaWldaWadaqaamaaqahabaGaem4qam0aaSbaaSqaaiabdMgaPbqabaGccqGGOaakcqWGQbGAcqGGPaqkcqGHxdaTcqWGTbqBcqWGHbqycqWG4baEcqWGFbWxcqWGZbWCcqWGJbWycqWGVbWBcqWGYbGCcqWGLbqzcqGGOaakcqWGQbGAcqGGPaqkaSqaaiabdQgaQjabg2da9iabigdaXaqaaiabd6gaUbqdcqGHris5aaGccaGLBbGaayzxaaGaaCzcaiaaxMaadaqadaqaaiabiodaZaGaayjkaiaawMcaaaqaaiabicdaWiabgsMiJkabd2gaTjabdggaHjabdsha0jabc+faFjabdohaZjabdMgaPjabd2gaTjabgsMiJkabigdaXaaaaa@87C9@

A subset of 164 PWMs representative of the major families of human transcription factors contained in TRANSFAC 6.0 was employed in this study.

### Optimization of TRANSFAC thresholds

When promoter sequences are analyzed for potential transcription factor binding sites, they are selected based on their similarity to TRANSFAC position weight matrices. This similarity is represented by its matrix similarity (mat_sim, Equation 3). By setting a threshold, only potential binding sites with an equal or larger similarity are selected. Because each position weight matrix is of differing inherent degeneracy, an optimized matrix threshold was generated for each matrix to provide an alternative method to minimize the number of potential false positive binding sites. To optimize the matrix thresholds, 1,000,000 bases of random sequence were analyzed with each matrix at intervals of successively higher matrix thresholds. The optimum threshold was arbitrarily defined for each matrix to be the point at which the matrix was detected as scoring only one binding site per 1000 bp of the random DNA.

### Microarray analysis

Analysis was performed on previously published microarray data [11] to generate a list of genes separated into groups based on specific steady-state mRNA expression levels. In these studies, the Jurkat human T-cell line was stimulated with phorbol ester and ionomycin. mRNA was isolated from cells at 0, 1, 2, 6, 12, and 24 hours after stimulation. The prefiltered hybridization signals (provided by Dieh et al [11]) were normalized and filtered to remove any spots marked as "bad" on any of the twelve arrays. The ratio expression data was then log transformed and standardized to a control set of hybridization signals from mRNA isolated from untreated Jurkat T-cells at each time point. The promoter regions (defined as 1200 bp upstream and 200 bp downstream from the transcription start site) of the resulting list of Unigene clusters were then retrieved using the ProSpector website. UniGene clusters for which no sequence could be found were discarded in this step. A final list of 7298 UniGene clusters and promoter regions remained. For analysis of gene expression data, the method of K-means clustering was used [54]. The optimum number of clusters for the data set (four) was determined using the method described by Davies and Bouldin [55].

### Promoter analysis

The genes analyzed in the microarray analysis were also analyzed for putative transcription factor binding using the ProSpector website . The gene promoter regions spanning 1200 bp upstream of transcription start and 200 bp downstream of transcription start were analyzed with all 164 position weight matrices. The analysis was performed twice: once applying a common matrix threshold of 0.75 and again with the optimized matrix thresholds. In both analyses, a threshold of 1 for the core similarity was used. The results of the analysis were analyzed by segregating the genes based on the number of each position weight matrix that scored a binding site in the promoter. As in the microarray analysis, the method of Davies and Bouldin was used to determine the optimal number of clusters according to promoter composition. K-means clustering was used to segregate the genes. In the analysis with a blanket 0.75 matrix threshold, the data was found through Davies-Bouldin analysis to separate into 16 groups. These clusters remained stable after the introduction of normalized Gaussian noise up to two fold standard deviation. Seven of the groups were small, containing 1 to 4 genes, and were discarded as outliers. The final result was 9 groups. The analysis was also conducted using optimized thresholds (see above). This analysis segregated into 6 groups. Again, groups with 4 or less genes were discarded as outliers leaving 4 groups derived from the optimized threshold TFBS analysis. For each analysis, clustered genes were analyzed using ANOVA to determine the transcription factor binding site motifs most significant in differentiating the clusters. Principal component analysis (PCA), ANOVA (one-way) and K-means clustering analysis were performed using Partek Pro 5.0 (Partek Corp.).

### Gene ontology

The GoMiner gene ontology tool was used to rank and categorize the gene ontology terms that were more significantly enriched beyond random assignment in each gene cluster [16]. Gene ontology terms were ranked according to *p*-values (Fisher's Exact T-test) generated by GoMiner [16]. Testing comparing ten random gene groups (1000 each) showed only random grouping of ontology terms versus the total population (data not shown). GoMiner was also used to identify those ontology terms that were enriched in the genes groups encoding the transcription factors known to associate with the most significant TFBSs identified in each respective cluster by ANOVA analysis. Significance testing for shared gene cluster ontology (GCO) terms and transcription factor ontology (TFO) terms was done by estimating the random probability of observing significant ontology terms in both TFO and GCO. The number of TFO terms were considered to be N, of which n are significant, and there are m significant GCO's found in TFO list of which k are also significant TFO's. The probability of obtaining k terms was tested if n values are randomly drawn from all TFO terms in which m are GCO's. The random process describing the null-hypothesis (no preferential overlap of GO terms) is described by the hypergeometric distribution which can be calculated by the phyper function of the R-Statistical software [56].

### Implementation

The ProSpector promoter retrieval and annotation tool is available for open access at . ProSpector is compatible with Internet Explorer, Mozilla, Firefox, Opera and Safari.

## Authors' contributions

MCM conceived of, designed, and implemented the ProSpector promoter annotation tool, carried out the promoter and ontology term analysis and assisted in the drafting of the manuscript. RT carried out statistics, promoter and ontology term analysis and assisted in the drafting of the manuscript. WC, IC, WJF, IM and CMH provided advice and assisted in the preparation of the manuscript. GVRC provided advice and performed much of the statistical analysis. KG conceived of the project, provided advice and opinions essential to the development of the analysis and helped draft the manuscript. All authors read and approved the final manuscript.

## Supplementary Material

Additional file 1 Supplemental Table 2, ontology terms associated with the 4 clusters in Figure 2 Click here for file

Additional file 2 Supplemental Table 3, representative genes from Clusters one, six and thirteen Click here for file
