# Supplementary material for: Human promoter genomic composition demonstrates non-random groupings that reflect general cellular function
Source: BMC Bioinformatics. 2005 Oct 18;6:259. doi: 10.1186/1471-2105-6-259 (PMC1274301; doi:10.1186/1471-2105-6-259)
Supplement: Additional file 1 — Supplemental Table 2, ontology terms associated with the 4 clusters in Figure 2 [file 1471-2105-6-259-S1.pdf]

Cluster One *(2997)*

p-Value	Ontology Term
0.0002	plasma protein
0.0002	complement activity
0.0005	humoral defense mechanism (sensu Vertebrata)
0.0008	complement activation
0.0008	intracellular
0.001	RNA polymerase II transcription factor activity
0.0012	complement activation, alternative pathway
0.0012	interleukin-1, Type I, activating receptor activity
0.0012	interleukin-1 receptor activity
0.0012	interleukin-1, Type I, activating binding activity
0.0012	interleukin-1 binding activity
0.0013	defense response
0.0018	humoral immune response
0.002	immune response
0.0046	oogenesis
0.0049	response to external stimulus
0.0066	glycine hydroxymethyltransferase activity
0.0091	specific RNA polymerase II transcription factor activity
0.0102	response to biotic stimulus
0.0113	cell growth and/or maintenance
0.0117	neurogenesis
0.0119	cell adhesion molecule activity
0.0133	coenzymes and prosthetic group metabolism
0.0136	ligand-dependent nuclear receptor activity
0.0136	steroid hormone receptor activity
0.0145	ligand-regulated transcription factor activity
0.0178	defense/immunity protein activity
0.0179	nucleus
0.0201	morphogenesis
0.0225	molecular_function
0.0232	cellular process
0.0254	secretory vesicle
0.0254	antimicrobial peptide activity
0.0257	response to pest/pathogen/parasite
0.0265	chemotaxis
0.0265	taxis
0.0274	cell
0.0301	cell migration
0.031	transmembrane receptor protein kinase activity
0.0342	coenzymes and prosthetic group biosynthesis

Cluster Three *(631)*

p-Value	Ontology Term
0.0001	actin filament-based process
0.0002	response to biotic stimulus
0.0004	actin cytoskeleton organization and biogenesis
0.0009	phorbol ester receptor activity
0.0009	protein kinase C activity
0.0011	defense response
0.0015	immune response
0.0023	protein tyrosine kinase activity
0.0029	GDP-dissociation inhibitor activity
0.0038	cell communication
0.0041	response to pest/pathogen/parasite
0.0041	protein serine/threonine kinase activity
0.0053	defense/immunity protein activity
0.0055	enzyme activator activity
0.0058	protein kinase activity
0.0067	actin cytoskeleton reorganization
0.0067	RAN protein binding activity
0.0069	cAMP-dependent protein kinase activity
0.0069	cyclic-nucleotide dependent protein kinase activity
0.0074	protein amino acid phosphorylation
0.0076	cellular process
0.0082	actin binding activity
0.0085	galactosylgalactosylxylosylprotein 3-beta-glucuronosyltransferase activity
0.0085	actin modulating activity
0.0085	actin monomer binding activity
0.0085	regulation of actin polymerization and/or depolymerization
0.0086	response to wounding
0.0096	protein binding activity
0.0103	small GTPase mediated signal transduction
0.0109	intracellular signaling cascade
0.011	cytoskeletal protein binding activity
0.0125	protein C-terminus binding activity
0.0138	protein kinase CK2 activity
0.0143	phosphotransferase activity, alcohol group as acceptor
0.0146	response to external stimulus
0.0163	signal transduction
0.0171	phosphorylation
0.0182	G-protein coupled receptor activity

Cluster Four *(1550)*

p-Value	Ontology Term
0.0002	plasma protein
0.0002	complement activity
0.0005	humoral defense mechanism (sensu Vertebrata)
0.0008	complement activation
0.0008	intracellular
0.001	RNA polymerase II transcription factor activity
0.0012	complement activation, alternative pathway
0.0012	interleukin-1, Type I, activating receptor activity
0.0012	interleukin-1 receptor activity
0.0012	interleukin-1, Type I, activating binding activity
0.0012	interleukin-1 binding activity
0.0013	defense response
0.0018	humoral immune response
0.002	immune response
0.0046	oogenesis
0.0049	response to external stimulus
0.0066	glycine hydroxymethyltransferase activity
0.0091	specific RNA polymerase II transcription factor activity
0.0102	response to biotic stimulus
0.0113	cell growth and/or maintenance
0.0117	neurogenesis
0.0119	cell adhesion molecule activity
0.0133	coenzymes and prosthetic group metabolism
0.0136	ligand-dependent nuclear receptor activity
0.0136	steroid hormone receptor activity
0.0145	ligand-regulated transcription factor activity
0.0178	defense/immunity protein activity
0.0179	nucleus
0.0201	morphogenesis
0.0225	molecular_function
0.0232	cellular process
0.0254	secretory vesicle
0.0254	antimicrobial peptide activity
0.0257	response to pest/pathogen/parasite
0.0265	chemotaxis
0.0265	taxis
0.0274	cell
0.0301	cell migration
0.031	transmembrane receptor protein kinase activity
0.0342	coenzymes and prosthetic group biosynthesis

Cluster Six *(2117)*

p-Value	Ontology Term
<0.0001	immune response
<0.0001	nucleobase, nucleoside, nucleotide and nucleic acid metabolism
<0.0001	intracellular
<0.0001	metabolism
0.0001	defense response
0.0001	G-protein coupled receptor protein signaling pathway
0.0001	response to pest/pathogen/parasite
0.0002	transcription from Pol II promoter
0.0003	nucleus
0.0003	extracellular
0.0004	humoral immune response
0.0008	rhodopsin-like receptor activity
0.0009	response to external stimulus
0.0013	G-protein coupled receptor activity
0.002	cell surface receptor linked signal transduction
0.0021	acid-D-amino acid ligase activity
0.003	nucleoplasm
0.0038	perception of external stimulus
0.0039	transferase activity, transferring phosphorus-containing groups
0.0041	phospholipase activity
0.0041	chromatin remodeling complex
0.0048	26S proteasome
0.005	extracellular space
0.0056	ubiquitin-protein ligase activity
0.006	response to wounding
0.0061	receptor activity
0.0064	purine nucleotide metabolism
0.0069	regulation of transcription from Pol II promoter
0.0075	DNA repair
0.0076	integral to membrane
0.0077	sensory perception
0.0078	kinase activity
0.008	xenobiotic metabolism
0.008	response to xenobiotic stimulus
0.0083	humoral defense mechanism (sensu Vertebrata)
0.0085	non-membrane spanning protein tyrosine phosphatase activity
0.0093	DNA metabolism
0.0096	serine-type endopeptidase activity
0.0103	nucleobase, nucleoside, nucleotide kinase activity
0.0108	small GTPase mediated signal transduction

Table Two (supplemental)
