# Supplementary material for: Human promoter genomic composition demonstrates non-random groupings that reflect general cellular function
Source: BMC Bioinformatics. 2005 Oct 18;6:259. doi: 10.1186/1471-2105-6-259 (PMC1274301; doi:10.1186/1471-2105-6-259)
Supplement: Additional file 2 — Supplemental Table 3, representative genes from Clusters one, six and thirteen [file 1471-2105-6-259-S2.pdf]

Representative Genes from Cluster One

**Gene**[a]	**Gene Function**[b]	**Empirical Verification for E2F regulation**[c]
ASK	DNA synthesis and replication	[1,2]
BCL2	Apoptosis	[3]
CDC14B	DNA synthesis and replication	[1]
CDC25C	Cell cycle G1	
CDC27	DNA synthesis and replication	
CDC6	DNA synthesis and replication	
CDC7L1	DNA synthesis and replication	
CDCA7	Cell cycle	
CDKN1A	Negative regulator of cell cycle	[4]
CDKN2A	Negative regulator of cell cycle	[5]
CKS1	Cell cycle S/G2	[6]
CTGF	Regulation of cell growth	[3]
EED	Development	[3,7]
FEN1	DNA damage repair	[6,7]
HEC	Cell cycle S/G2	[2]
HOXA5	Development	[1]
KIF4A	Cell Cycle S/G2	[2,6]
MAD2L1	Checkpoints	[2]
MCL1	Anti-apoptosis	[4]
MCM7	DNA synthesis and replication	[6,7]
ORC1L	DNA synthesis and replication	[2]
PCNA	DNA synthesis and replication	[1,6,7]
PLK	Cell Cycle S/G2	[6]
PRIM2A	DNA synthesis and replication	[1,2]
RB1	Checkpoints	[2,6]
RFC1	DNA synthesis and replication	[1,6]
RFC4	DNA synthesis and replication	[2,7]
RRM1	DNA synthesis and replication	[2,6]
SMC2L1	Cell Cycle S/G2	[2]
SMC4L1	Cell Cycle S/G2	[2]
TOP2A	DNA synthesis and replication	[6,7]
TP53	Checkpoints	[2,6]

[a]HUGO approved gene symbol
[b]As annotated in [8]
[c]Partially compiled from references in [8]

Table Three (supplemental)

Reference List

1. Stanelle J, Stiewe T, Theseling CC, Peter M, Putzer BM: **Gene expression changes in response to E2F1 activation.** *Nucleic Acids Res* 2002, **30:** 1859-1867.

2. Ren B, Cam H, Takahashi Y, Volkert T, Terragni J, Young RA *et al*.: **E2F integrates cell cycle progression with DNA repair, replication, and G(2)/M checkpoints.** *Genes Dev* 2002, **16:** 245-256.

3. Muller H, Bracken AP, Vernell R, Moroni MC, Christians F, Grassilli E *et al*.: **E2Fs regulate the expression of genes involved in differentiation, development, proliferation, and apoptosis.** *Genes Dev* 2001, **15:** 267-285.

4. Gartel AL, Najmabadi F, Goufman E, Tyner AL: **A role for E2F1 in Ras activation of p21(WAF1/CIP1) transcription.** *Oncogene* 2000, **19:** 961-964.

5. Berkovich E, Lamed Y, Ginsberg D: **E2F and Ras synergize in transcriptionally activating p14ARF expression.** *Cell Cycle* 2003, **2:** 127-133.

6. Ishida S, Huang E, Zuzan H, Spang R, Leone G, West M *et al*.: **Role for E2F in control of both DNA replication and mitotic functions as revealed from DNA microarray analysis.** *Mol Cell Biol* 2001, **21:** 4684-4699.

7. Polager S, Kalma Y, Berkovich E, Ginsberg D: **E2Fs up-regulate expression of genes involved in DNA replication, DNA repair and mitosis.** *Oncogene* 2002, **21:** 437-446.

8. Bracken AP, Ciro M, Cocito A, Helin K: **E2F target genes: unraveling the biology.** *Trends Biochem Sci* 2004, **29:** 409-417.

Representative Genes from Cluster 6

**Gene**[a]	**Gene Function**[b]	**Empirical Verification for kappa B regulation**[b]
AMH	Anti-Mullerian hormone	[1]
EPO	Erythropoietin	[2]
ETR101	TPA-inducible, Jun-like transcription factor	[3]
FOS	Proto-oncogene	[4]
JUNB	Proto-oncogene	[5]
NFKB2	NF-kB p100 precursor	[6]
POMC	Proopiomelanocortin	[7]
PTGIS	Prostaglandin synthase	[8]
IL11	Interleukin-11	[9]
COL1A2	Collagen, type I, alpha 2	[10]
COL7A1	Collagen, type VII, alpha 1	[11]
CYP1A1	Cytochrome p450	[12]
ICAM1	Intracellular adhesion molecule 1	[13]
TGFB1	Transforming growth factor, beta 1	[14]
TNF	Tumor necrosis factor	[15,16]
CCND1	Cyclin D1	[17]
CCND2	Cyclin D2	[18]

[a]HUGO gene symbol
[b]Partially complied from http://people.bu.edu/gilmore/nf-kb/target/index.html

Reference List

1. Lukas-Croisier C, Lasala C, Nicaud J, Bedecarras P, Kumar TR, Dutertre M, Matzuk MM, Picard JY, Josso N, Rey R. **Follicle-stimulating hormone increases testicular Anti-Mullerian hormone (AMH) production through sertoli cell proliferation and a nonclassical cyclic adenosine 5'-monophosphate-mediated activation of the AMH Gene**. Mol Endocrinol. 2003 Apr;17(4):550-61. Epub 2003 Jan 2.

2. Figueroa YG, Chan AK, Ibrahim R, Tang Y, Burow ME, Alam J, Scandurro AB, Beckman BS. **NF-kappaB plays a key role in hypoxia-inducible factor-1-regulated erythropoietin gene expression.** Exp Hematol. 2002 Dec;30(12):1419-27.

3. Zhou A, Scoggin S, Gaynor RB, Williams NS. **Identification of NF-kappa B-regulated genes induced by TNFalpha utilizing expression profiling and RNA interference.** Oncogene. 2003 Apr 3;22(13):2054-64. Erratum in: Oncogene. 2004 Dec 16;23(58):9447.

4. Li Y, Kim IC, Kim YJ, Kim MK, Yoon YD, Lee YS, Lee JS. **Cloning and sequence analysis of the self-fertilizing fish Rivulus marmoratus immediate early gene c-fos.** Mar Environ Res. 2004 Aug-Dec;58(2-5):681-5.

5. Brown RT, Ades IZ, Nordan RP. **An acute phase response factor/NF-kappa B site downstream of the junB gene that mediates responsiveness to interleukin-6 in a murine plasmacytoma.** J Biol Chem. 1995 Dec 29;270(52):31129-35.

6. Lombardi L, Ciana P, Cappellini C, Trecca D, Guerrini L, Migliazza A, Maiolo AT, Neri A. **Structural and functional characterization of the promoter regions of the NFKB2 gene.** Nucleic Acids Res. 1995 Jun 25;23(12):2328-36.

7. Karalis KP, Venihaki M, Zhao J, van Vlerken LE, Chandras C. **NF-kappaB participates in the corticotropin-releasing, hormone-induced regulation of the pituitary proopiomelanocortin gene.** J Biol Chem. 2004 Mar 19;279(12):10837-40. Epub 2004 Jan 6.

8. Yokoyama C, Yabuki T, Inoue H, Tone Y, Hara S, Hatae T, Nagata M, Takahashi EI, Tanabe T. **Human gene encoding prostacyclin synthase (PTGIS): genomic organization, chromosomal localization, and promoter activity.** Genomics. 1996 Sep 1;36(2):296-304.

9. Bitko V, Velazquez A, Yang L, Yang YC, Barik S. **Transcriptional induction of multiple cytokines by human respiratory syncytial virus requires activation of NF-kappa B and is inhibited by sodium salicylate and aspirin.** Virology. 1997 Jun 9;232(2):369-78.

10. Kouba DJ, Chung KY, Nishiyama T, Vindevoghel L, Kon A, Klement JF, Uitto J, Mauviel A. **Nuclear factor-kappa B mediates TNF-alpha inhibitory effect on alpha 2(I) collagen (COL1A2) gene transcription in human dermal fibroblasts.** J Immunol. 1999 Apr 1;162(7):4226-34.

11. Kon A, Vindevoghel L, Kouba DJ, Fujimura Y, Uitto J, Mauviel A. **Cooperation between SMAD and NF-kappaB in growth factor regulated type VII collagen gene expression.** Oncogene. 1999 Mar 11;18(10):1837-44.

12. Ke S, Rabson AB, Germino JF, Gallo MA, Tian Y. **Mechanism of suppression of cytochrome P-450 1A1 expression by tumor necrosis factor-alpha and lipopolysaccharide.** J Biol Chem. 2001 Oct 26;276(43):39638-44. Epub 2001 Jul 24.

13. Voisard R, Huber N, Baur R, Susa M, Ickrath O, Both A, Koenig W, Hombach V. **Different effects of antisense RelA p65 and NF-kappaB1 p50 oligonucleotides on the nuclear factor-kappaB mediated expression of ICAM-1 in human coronary endothelial and smooth muscle cells.** BMC Mol Biol. 2001;2(1):7. Epub 2001 Aug 8.

14. Perez JR, Higgins-Sochaski KA, Maltese JY, Narayanan R. **Regulation of adhesion and growth of fibrosarcoma cells by NF-kappa B RelA involves transforming growth factor beta.** Mol Cell Biol. 1994 Aug;14(8):5326-32.

15. Shakhov AN, Collart MA, Vassalli P, Nedospasov SA, Jongeneel CV. **Kappa B-type enhancers are involved in lipopolysaccharide-mediated transcriptional activation of the tumor necrosis factor alpha gene in primary macrophages.** J Exp Med. 1990 Jan 1;171(1):35-47.

16. Collart MA, Baeuerle P, Vassalli P. **Regulation of tumor necrosis factor alpha transcription in macrophages: involvement of four kappa B-like motifs and of constitutive and inducible forms of NF-kappa B.** Mol Cell Biol. 1990 Apr;10(4):1498-506.

17. Romieu-Mourez R, Kim DW, Shin SM, Demicco EG, Landesman-Bollag E, Seldin DC, Cardiff RD, Sonenshein GE. **Mouse mammary tumor virus c-rel transgenic mice develop mammary tumors.** Mol Cell Biol. 2003 Aug;23(16):5738-54.

18. Piatelli MJ, Wardle C, Blois J, Doughty C, Schram BR, Rothstein TL, Chiles TC. **Phosphatidylinositol 3-kinase-dependent mitogen-activated protein/extracellular signal-regulated kinase kinase 1/2 and NF-kappa B signaling pathways are required for B cell antigen receptor-mediated cyclin D2 induction in mature B cells.** J Immunol. 2004 Mar 1;172(5):2753-62.

Representative Genes from Cluster 13

**Gene**[a]	**Gene Function**[b]	**Empirical Verification, of Sp1, EGR2 or AHR regulation**[c]
MYBL2	Regulation of transcription	[1]
RBL1	Regulation of transcription	[2]
SLC2A1	Transporter activity	[3]
SYN47	Regulation of transcription	[4]
TAO1	Regulation of cell growth	[4]
RPL10	Protein biosynthesis	[5][c]
RPL13	Protein biosynthesis	[5][c]
RPL18	Protein biosynthesis	[5][c]
RPL18A	Structural constituent of ribosome	[5][c]
RPL27A	Cytosolic large ribosomal subunit	[5][c]
RPL28	Cytosolic large ribosomal subunit	[5][c]
RPL35	Structural constituent of ribosome	[5][c]
RPS13	Structural constituent of ribosome	[5][c]
RPS28	Structural constituent of ribosome	[5][c]
CDKN1B	Cyclin-dependent protein kinase inhibitor	[6]
TCEB3	Transcriptional elongation regulator activity	[4]
HMG2	Nuclear chromosome	[7]
SMPD1	Signal transduction	[8]
SCTR	Receptor activity	[9]
PKM2	Glycolysis	[10]
PKD2	Cation channel activity	[11]
P2RX4	Receptor activity	[12]
NF2	Structural molecule activity	[13]
MXI1	Regulation of transcription	[14]
KRT19	Structural molecule activity	[15]
IGFBP3	Regulation of cell growth	[16]
IGFBP2	Regulation of cell growth	[17]
ID4	Transcription corepressor activity	[18]
HOXB7	Regulation of transcription	[19]
GSTP1	Metabolism	[20]
G22P1	Helicase activity	[21]
FMR1	mRNA binding	[22]
FGFR4	Receptor activity	[23]
CCND3	Regulation of cell cycle	[24]
BIRC5	Cysteine protease inhibitor activity	[25]

[a]HUGO approved gene symbol
[b]Extracted from www.genecards.org
[c]Computational corroboration

Reference List

1. Sala A, Saitta B, De Luca P, Cervellera MN, Casella I, Lewis RE, Watson R, Peschle C. **B-MYB transactivates its own promoter through SP1-binding sites.** Oncogene 1999 Feb 11;18(6):1333-9.
2. Voorhoeve PM, Watson RJ, Farlie PG, Bernards R, Lam EW. **Rapid dephosphorylation of p107 following UV irradiation.** Oncogene 1999 Jan 21;18(3):679-88.
3. Santalucía T, Boheler KR, Brand NJ, Sahye U, Fandos C, Viñals F, Ferré J, Testar X, Palacín M, Zorzano A. **Factors involved in GLUT-1 glucose transporter gene transcription in cardiac muscle.** J Biol Chem 1999 Jun 18;274(25):17626-34.
4. Nagarajan R, Svaren J, Le N, Araki T, Watson M, Milbrandt J. **EGR2 mutations in inherited neuropathies dominant-negatively inhibit myelin gene expression.** Neuron 2001 May 1;30(2):355-68.
5. Perry RP. **The architecture of mammalian ribosomal protein promoters.** BMC Evol Biol. 2005 Feb 13;5(1):15.
6. Kolluri SK, Weiss C, Koff A, Göttlicher M. **p27(Kip1) induction and inhibition of proliferation by the intracellular Ah receptor in developing thymus and hepatoma cells.** Genes Dev 1999 Jul 1;13(13):1742-53.
7. Krynetski EY, Krynetskaia NF, Bianchi ME, Evans WE. **A Nuclear Protein Complex Containing High Mobility Group Proteins B1 and B2, Heat Shock Cognate Protein 70, ERp60, and Glyceraldehyde-3- Phosphate Dehydrogenase Is Involved in the Cytotoxic Response to DNA Modified by Incorporation of Anticancer Nucleoside Analogues.** Cancer Res 2003 Jan 1;63(1):100-6.
8. Langmann T, Buechler C, Ries S, Schaeffler A, Aslanidis C, Schuierer M, Weiler M, Sandhoff K, de Jong PJ, Schmitz G. **Transcription factors Sp1 and AP-2 mediate induction of acid sphingomyelinase during monocytic differentiation.** J Lipid Res. 1999 May;40(5):870-80.
9. Pang RT, Lee LT, Ng SS, Yung WH, Chow BK. **CpG methylation and transcription factors Sp1 and Sp3 regulate the expression of the human secretin receptor gene.** Mol Endocrinol. 2004 Feb;18(2):471-83. Epub 2003 Nov 26.
10. Yamada K, Tanaka T, Miyamoto K, Noguchi T. **Sp family members and nuclear factor-Y cooperatively stimulate transcription from the rat pyruvate kinase M gene distal promoter region via their direct interactions.** J Biol Chem. 2000 Jun 16;275(24):18129-37.
11. Lantinga-van Leeuwen IS, Leonhard WN, Dauwerse H, Baelde HJ, van Oost BA, Breuning MH, Peters DJ. **Common regulatory elements in the polycystic kidney disease 1 and 2 promoter regions.** Eur J Hum Genet. 2005 May;13(5):649-59.

12. Korenaga R, Yamamoto K, Ohura N, Sokabe T, Kamiya A, Ando J. **Sp1-mediated downregulation of P2X4 receptor gene transcription in endothelial cells exposed to shear stress.** Am J Physiol Heart Circ Physiol. 2001 May;280(5):H2214-21.
13. Chang LS, Akhmametyeva EM, Wu Y, Zhu L, Welling DB. **Multiple transcription initiation sites, alternative splicing, and differential polyadenylation contribute to the complexity of human neurofibromatosis 2 transcripts.** Genomics. 2002 Jan;79(1):63-76.
14. Benson LQ, Coon MR, Krueger LM, Han GC, Sarnaik AA, Wechsler DS. **Expression of MXI1, a Myc antagonist, is regulated by Sp1 and AP2.** J Biol Chem. 1999 Oct 1;274(40):28794-802.
15. Brembeck FH, Rustgi AK. **The tissue-dependent keratin 19 gene transcription is regulated by GKLF/KLF4 and Sp1.** J Biol Chem. 2000 Sep 8;275(36):28230-9.
16. Walker GE, Wilson EM, Powell D, Oh Y. **Butyrate, a histone deacetylase inhibitor, activates the human IGF binding protein-3 promoter in breast cancer cells: molecular mechanism involves an Sp1/Sp3 multiprotein complex.** Endocrinology. 2001 Sep;142(9):3817-27.
17. Simmen RC, Zhang XL, Michel FJ, Min SH, Zhao G, Simmen FA. **Molecular markers of endometrial epithelial cell mitogenesis mediated by the Sp/Kruppel-like factor BTEB1.** DNA Cell Biol. 2002 Feb;21(2):115-28.
18. Pagliuca A, Cannada-Bartoli P, Lania L. **A role for Sp and helix-loop-helix transcription factors in the regulation of the human Id4 gene promoter activity.** J Biol Chem. 1998 Mar 27;273(13):7668-74.
19. Meccia E, Bottero L, Felicetti F, Peschle C, Colombo MP, Care A. **HOXB7 expression is regulated by the transcription factors NF-Y, YY1, Sp1 and USF-1.** Biochim Biophys Acta. 2003 Apr 15;1626(1-3):1-9.
20. Moffat GJ, McLaren AW, Wolf CR. **Sp1-mediated transcriptional activation of the human Pi class glutathione S-transferase promoter.** J Biol Chem. 1996 Jan 12;271(2):1054-60.
21. Hosoi Y, Watanabe T, Nakagawa K, Matsumoto Y, Enomoto A, Morita A, Nagawa H, Suzuki N. **Up-regulation of DNA-dependent protein kinase activity and Sp1 in colorectal cancer.** Int J Oncol. 2004 Aug;25(2):461-8.
22. Smith KT, Coffee B, Reines D. **Occupancy and synergistic activation of the FMR1 promoter by Nrf-1 and Sp1 in vivo.** Hum Mol Genet. 2004 Aug 1;13(15):1611-21. Epub 2004 Jun 2.
23. Yu S, Asa SL, Ezzat S. **Fibroblast growth factor receptor 4 is a target for the zinc-finger transcription factor Ikaros in the pituitary.** Mol Endocrinol. 2002 May;16(5):1069-78.
24. Yang M, Nomura H, Hu Y, Kaneko S, Kaneko H, Tanaka M, Nakashima K. **Prolactin-induced expression of TATA-less cyclin D3 gene is mediated by Sp1 and AP2.** Biochem Mol Biol Int. 1998 Jan;44(1):51-8.
25. Li F, Altieri DC. **The cancer antiapoptosis mouse survivin gene: characterization of locus and transcriptional requirements of basal and cell cycle-dependent expression.** Cancer Res. 1999 Jul 1;59(13):3143-51.
